# Factors Influencing Primary Care Practitioners’ Cancer Screening Recommendations for Older Adults: a Systematic Review

**DOI:** 10.1007/s11606-023-08213-4

**Published:** 2023-05-04

**Authors:** Jenna Smith, Rachael H. Dodd, Karen M. Gainey, Vasi Naganathan, Erin Cvejic, Jesse Jansen, Kirsten J. McCaffery

**Affiliations:** 1 Wiser Healthcare, Sydney School of Public Health, Faculty of Medicine and Health, The University of Sydney, Sydney, NSW Australia; 2 Sydney Health Literacy Lab, Sydney School of Public Health, Faculty of Medicine and Health, The University of Sydney, Sydney, NSW Australia; 3 Sydney School of Public Health, Faculty of Medicine and Health, The University of Sydney, Sydney, NSW 2006 Australia; 4 The Daffodil Centre, a joint venture between Cancer Council NSW and The University of Sydney, Faculty of Medicine and Health, Sydney, NSW Australia; 5 Concord Clinical School, Faculty of Medicine and Health, The University of Sydney, Sydney, NSW Australia; 6 Centre for Education and Research On Ageing, Department of Geriatric Medicine, Concord Repatriation Hospital, Sydney, NSW Australia; 7 School for Public Health and Primary Care, Faculty of Health, Medicine and Life Sciences, Maastricht University, Maastricht, The Netherlands

## Abstract

**Background:**

Primary care practitioners (PCPs) play a key role in cancer screening decisions for older adults (≥ 65 years), but recommendations vary by cancer type and jurisdiction.

**Purpose:**

To examine the factors influencing PCPs’ recommendations for breast, cervical, prostate, and colorectal cancer screening for older adults.

**Data Sources:**

MEDLINE, Pre-Medline, EMBASE, PsycINFO, and CINAHL, searched from 1 January 2000 to July 2021, and citation searching in July 2022.

**Study Selection:**

Assessed factors influencing PCPs’ breast, prostate, colorectal, or cervical cancer screening decisions for older adults’ (defined either as ≥ 65 years or < 10-year life expectancy).

**Data Extraction:**

Two authors independently conducted data extraction and quality appraisal. Decisions were crosschecked and discussed where necessary.

**Data Synthesis:**

From 1926 records, 30 studies met inclusion criteria. Twenty were quantitative, nine were qualitative, and one used a mixed method design. Twenty-nine were conducted in the USA, and one in the UK. Factors were synthesized into six categories: patient demographic characteristics, patient health characteristics, patient and clinician psycho-social factors, clinician characteristics, and health system factors. Patient preference was most reported as influential across both quantitative and qualitative studies. Age, health status, and life expectancy were also commonly influential, but PCPs held nuanced views about life expectancy. Weighing benefits/harms was also commonly reported with variation across cancer screening types. Other factors included patient screening history, clinician attitudes/personal experiences, patient/provider relationship, guidelines, reminders, and time.

**Limitations:**

We could not conduct a meta-analysis due to variability in study designs and measurement. The vast majority of included studies were conducted in the USA.

**Conclusions:**

Although PCPs play a role in individualizing cancer screening for older adults, multi-level interventions are needed to improve these decisions. Decision support should continue to be developed and implemented to support informed choice for older adults and assist PCPs to consistently provide evidence-based recommendations.

**Registration:**

PROSPERO CRD42021268219.

**Funding Source:**

NHMRC APP1113532.

**Supplementary Information:**

The online version contains supplementary material available at 10.1007/s11606-023-08213-4.

## INTRODUCTION

Deciding whether it is worthwhile to advise older adults (aged ≥ 65 years) to continue or stop cancer screening is not straightforward for primary care practitioners (PCPs). At a population level, there is evidence that the benefit/harm trade-off of cancer screening becomes less favorable in older adults. The decision also becomes increasingly complex given competing mortality risks, increasing risks of harm from the screening cascade (follow-up testing, false positive results) and harms from overdiagnosis.^1^ Moreover, the chance of benefiting at an individual level does not depend on age, but rather the life expectancy and health status of a person, suggesting screening decisions should be individualized.^2^

Cancer screening recommendations for older adults vary by jurisdiction and cancer type. Many countries implement national breast, bowel, and cervical screening programs that target participation up to a certain age (e.g., 75 years), but it is unclear what role PCPs play in decision-making beyond these ages. In the USA, both researchers and guidelines from the U.S. Preventive Services Task Force have increasingly advocated for individualized screening decisions based on patient health, comorbidities, and prior screening history,^1–4^ in which PCPs play a key role. For example, many guidelines recommend against screening older adults with less than 10 years’ life expectancy.^1^ However, many older adults with limited life expectancy continue to screen^5^ and have limited knowledge of the potential harms of doing so.^6^

Understanding more about the evidence on the factors that influence PCPs’ decisions about continuing or stopping cancer screening in older adults is needed to inform the design of interventions to individualize screening for older adults and to identify areas for further research in this area. The aim of this systematic review was to examine the factors influencing PCPs’ recommendations for breast, cervical, prostate, and colorectal cancer screening for older adults. These cancer screening types were chosen given the international relevance of the problem of overscreening and low-value screening for all four types.^5,7–9^

## METHODS

### Protocol and Registration

The protocol for this review was registered with PROSPERO (CRD42021268219) and reporting is guided by the Preferred Reporting Items for Systematic Reviews and Meta-Analyses (PRISMA) checklist.^10^

### Search Strategy

A comprehensive search strategy previously developed in consultation with an academic librarian to explore patient-reported factors associated with older adults’ cancer screening decision-making^11^ was adapted by adding terms related to PCPs (see Supplementary Table I). Searches were conducted in Medline, Pre-Medline, EMBASE, PsycINFO, and CINAHL on 16th July 2021. After removing duplicates, two researchers (JS, RD) independently screened titles and abstracts for inclusion/exclusion using an eligibility checklist and disagreements were resolved via discussion. A backward citation search and forward citation search were conducted once decisions were finalized (14th July 2022).

### Inclusion Criteria

Studies were included if they:

- Empirically assessed clinician-reported factors influencing primary care practitioners’ (PCPs) recommendations for cancer screening for older adults, defined as either (1) adults who are aged ≥ 65 years or (2) adults with limited (< 10-year) life expectancy.
- Included PCPs (e.g., general practitioners, family medicine practitioners, primary care internists, primary care nurse practitioners, physician assistants) providing care to older adults or a sub-group of older adults.
- Assessed breast (mammography), cervical (Pap smear or human papillomavirus (HPV) test), prostate (prostate-specific antigen (PSA) testing), or colorectal (faecal occult blood testing (FOBT), faecal immunochemical test (FIT), or colonoscopy) cancer screening decisions in asymptomatic older patients; and
- Were quantitative, qualitative, or mixed-methods peer-reviewed publications, or theses.

Studies were excluded if they:

- Analyzed factors associated with retrospective screening, or assessed decision-making regarding diagnostic or surveillance tests; or
- Were reviews, editorials, commentaries, research letters, intervention studies or protocols.

### Quality Appraisal

Two researchers (JS, KG) independently assessed the quality of included studies using the Joanna Briggs Institute critical appraisal tools, which are checklists that can be used to determine the sources of bias in a study’s design, conduct, and analysis (Prevalence Studies Checklist for survey studies and Qualitative Research Checklist for interview and focus group studies).^12^ Different aspects of the methods and reporting of results in included studies were scored individually and then combined to determine an overall assessment of risk of bias (low, moderate, or high). See Supplementary Table II for more details.

### Data Extraction and Synthesis

Previous systematic reviews conducted by the research team and their colleagues were used to inform the development of a standardized data extraction template (Supplementary Table III).^11,13^ After piloting, two authors (JS, KG) independently extracted data and resolved any disagreements through discussion. Disagreements were evident for the categorization of factors, for example, whether fear of what the patient thought was a “patient psychosocial” or “clinician psychosocial” factor. These discrepancies were solved through input of all other co-authors. The data was narratively summarized. JS conducted a preliminary synthesis, which was continually refined throughout the writing process through input from all co-authors.

## RESULTS

**Figure 1:**
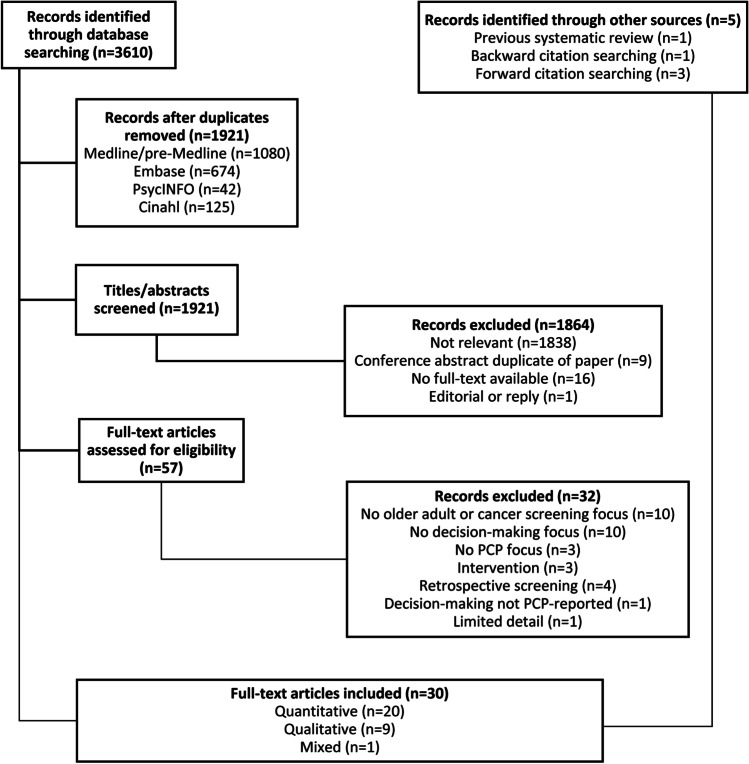
Flow diagram of included studies.

After removal of duplicates, 1921 titles and abstracts were screened for inclusion or exclusion, and 57 full texts were reviewed, of which 25 met inclusion criteria (Fig. 1). Five additional records were included: one identified in our review on patient-reported factors,^14^ three from forward citation searching,^15–17^ and one from backward citation searching.^18^ This led to the inclusion of 30 studies (20 quantitative, nine qualitative, and one mixed-methods).

Characteristics of included studies are summarized in Table 1. Eight studies included family and internal medicine specialties only;^22–25,30,35,41,42^ 21 included a combination of family/internal medicine and geriatricians, urologists, oncologists, and obstetrics and gynecology providers;^14–21,26–29,31–34,36–39,43^ and one included resident physicians.^40^ Most did not differentiate results across specialty types, except where reported. Thirteen studies examined breast screening;^14,15,17,18,23,26,29,30,32,39,42–44^ six examined colorectal;^21,24,25,31,34,40^ five examined prostate;^16,36–38,41^ one examined cervical;^19^ three examined a combination of breast, colorectal, and prostate;^20,27,33^ one examined breast and cervical;^22^ and one examined prostate and colorectal.^45^

**Table 1:** Characteristics of Included Studies

Study (author, year)	Country and Area	Study aim	Study design	Sample size	Description of sample	Cancer screening type	Factors included in study	Risk of bias
Austin 2021^17^	New York City (NYC), USA	To explore factors contributing to BCa screening overuse at one of the largest NYC ambulatory care settings and elicit potential strategies to reduce overuse	Qualitative, semi-structured interview	5	PCPs who provide care to women 70 + years and have experiential BCa screening knowledge and insights	Breast	Pt demographics and health Pt and clinician psycho-social Health system	Low
Boone 2018^19^	California, USA	Understand the motivations, beliefs, and circumstances which prompt PCPs to knowingly recommend continued cervical screening in low-risk women > 65 years, despite specific awareness of consensus guidelines to the contrary	Cross-sectional survey, self-reported reasons	1259	Family medicine physicians, obstetrician/gynecologists, primary care internists, PCP assistants, and primary care nurse practitioners	Cervical	Pt and clinician psycho-social Health system	Low
Enns 2021^20^	Maryland, USA	Understand similarities and differences in clinician decision-making for BCa, PCa, and CRC in older adults	Qualitative, semi-structured interviews	30	Internal medicine, family medicine, geriatrics, and medicine/pediatrics physicians	Breast Prostate Colorectal	Pt demographics and health Clinician psycho-social	Low
Haggstrom 2013^21^	USA	Characterize the extent to which PCPs modify their recommendations for CRC screening based upon patients’ increasing age and/or worsening comorbidity	Cross-sectional, vignettes	1266	General internal medicine, family medicine, and obstetrics-gynecology physicians	Colorectal	Pt demographics and health Clinician characteristics	Low
Heflin 2006^22^	USA	Examine the association of health status with physicians’ self-reported intentions to offer BCa screening and Pap smear to older women	Cross-sectional, vignettes	2003	PCPs drawn from the American Medical Association’s Physician Masterfile	Breast Cervical	Pt demographics and health Clinician characteristics	Low
Kadaoui 2012^23^	Canada	Describe physician practices regarding opportunistic screening for BCa in women 70 + years and identify determinants associated with prescribing BCa screening	Cross-sectional, vignettes	460	General practitioners practicing in Quebec	Breast	Pt demographics and health Clinician psycho-social	Low
Leach 2012^15^	USA	To consider the BCa screening recommendations of PCPs for women of various age and health status combinations	Cross-sectional, national survey, vignettes	1212	PCPs with an active medical license who were engaged in patient care as their main professional activity	Breast	Pt demographics and health Clinician characteristics	Low
Lewis 2013^24^	USA	Examine whether physicians elect to engage older patients in individualized decision-making for CRC screening	Cross-sectional, self-reported recommendation	276	Family physicians and general internists	Colorectal	Pt demographics and health Clinician characteristics	Low
Lewis 2009^25^	North Carolina, USA	Learn whether physicians employ individualized decision-making for CRC screening in older adults and to explore factors they believe are important to make these decisions	Qualitative; focus groups and semi-structured interviews	15	PCPs who supervise 1^st^ and 2^nd^ year students in the Introduction to Clinical Medicine Course at University of North Carolina	Colorectal	Pt demographics and health Pt and clinician psycho-social	Low
Radhakrishnan 2018^26^	USA	Examine whether attitudes/beliefs towards BCa screening were associated with BCa screening recommendations for women of different ages	Cross-sectional; self-reported recommendation	871	Internal medicine, family medicine, general practice physicians, and gynecologists	Breast	Clinician psycho-social	Low
Rowe 2021^16^	Chicago, USA	To explore perspectives and barriers to guideline adherence from clinicians who are over-screeners for PCa	Qualitative; semi-structured interviews	14	PCPs who were high-utilizers of PCa screening in men aged ≥ 76 years	Prostate	Pt and clinician psycho-social Health system	Low
Schoenborn 2020a^27^	USA	Examine PCPs’ decision-making on stopping cancer screening in specific older patients with limited life expectancy	Qualitative, semi-structured interviews	25	Clinicians who provided primary care to older adults	Breast Prostate Colorectal	Pt demographics and health Pt and clinician psycho-social Health system	Low
Siembida 2017^28^	USA	Examine the extent to which reminders have been deployed for BCa screening targeting older patients	Cross-sectional, self-reported recommendation	871	Internal medicine, family medicine, general practice physicians, and gynecologists	Breast	Health system	Low
Yasmeen 2012^29^	USA	Explore decisions regarding BCa screening in hypothetical clinical case scenarios; and predictors of recommendations for BCa screening in different age categories	Cross-sectional, vignettes	684	General internists, family physicians, and obstetricians and gynecologists	Breast	Clinician characteristics Health system	Low
Oshima 2021^30^	North Carolina, USA	Explore perspectives on screening mammography in older women, factors that influence these practices, as well as barriers to and facilitators of patient counselling	Qualitative; semi-structured interviews	10	Physicians and mid-level providers who self-reported participation in the care of women aged ≥ 75 years	Breast	Pt demographics and health Pt and clinician psycho-social Health system	Low-moderate
Park 2021^31^	Maryland, USA	Examine clinicians’ perceptions of different CRC screening test options in older adults	Qualitative; semi-structured interviews	30	Physicians, certified registered nurse practitioners, and physician assistants	Colorectal	Pt demographics and health Clinician psycho-social Health system	Low-moderate
Pollack 2018^32^	USA	Investigate whether experiences with patients, friends, colleagues and family members diagnosed with BCa were associated with providers' BCa screening recommendations	Cross-sectional, self-reported recommendation	871	Gynecologists and internal medicine, family medicine and general practice physicians	Breast	Clinician psycho-social	Low-moderate
Schoenborn 2020b^33^	USA	Understand clinicians’ views around overscreening and specifically around the current approach of using limited life expectancy to guide screening cessation	Qualitative, semi-structured interviews	30	Physicians, certified registered nurse practitioners, and physician assistants who cared for adults 65 +	Breast Prostate Colorectal	Pt demographics and health Pt and clinician psycho-social Health system	Low-moderate
Haas 2017^18^	USA	To directly examine the association of PCP beliefs about BCa screening effectiveness and recommendations for screening frequency with screening utilization	Cross-sectional, linked survey data	209	PCPs affiliated with the three primary care networks of breast cancer research centers	Breast	Clinician psycho-social	Moderate
Kahi 2009^34^	USA	Determine patient (comorbidity and prior screening history) and provider characteristics associated with recommending CRC screening for older patients	Cross-sectional, vignettes	183	Veteran affairs PCPs	Colorectal	Pt demographics and health Clinician characteristics	Moderate
Kistler 2018^35^	USA	Examine when PCPs would recommend low-value screening, as described by USPSTF recommendations on PCa and CRC screening in effect at the time of this study	Cross-sectional, vignettes	126	PCPs from family medicine or general internal medicine clinics	Prostate Colorectal	Pt demographics and health Pt and clinician psycho-social Health system	Moderate
Konety 2009^36^	USA	Develop recommendations for uniform approach to PCa screening in men > 75 years and determine whether attitudes toward continued screening has changed	Cross-sectional, self-reported recommendation	614	PCPs from family medicine or general internal medicine clinics	Prostate	Clinician characteristics	Moderate
Konety 2006^37^	Iowa, USA	Assess healthcare provider preferences regarding PCa screening for men 75 + years	Cross-sectional, self-reported recommendation	997	PCPs from internal medicine, geriatrics, family medicine, urology, medical oncology, radiation oncology and physician’s assistants	Prostate	Pt demographics and health Clinician characteristics	Moderate
Pollack 2012^38^	USA	Examine whether providers take age and life expectancy into account when ordering PCa screening and determine what barriers clinicians face when discontinuing screening	Cross-sectional; self-reported ordering	125	Individuals who provide primary care for adult male patients	Prostate	Pt demographics and health Pt psycho-social Health system	Moderate
Schonberg 2006^39^	USA	Explore decision-making and physician counselling of oldest-old women around mammography screening	Qualitative; semi-structured interviews	16	Physicians at academic primary care practice	Breast	Pt demographics and health Pt and clinician psycho-social Health system	Moderate
Walters 2011^14^	England	Establish whether PCPs are in favour of screening extension in the UK, to whom, and how it should be offered and how accurately they can assess which women may benefit	Mixed methods; cross-sectional, vignettes, semi-structured interviews	Surveys: 139 Interviews: 26	Healthcare professionals with expertise in the field of BCa management or elderly care	Breast	Pt demographics and health Pt and clinician psycho-social Clinician characteristics Health system	Moderate-high
Lewis 2008^40^	USA	Assess whether physicians use individualized approach for CRC screening recommendations in adults aged ≥ 75	Cross-sectional, vignettes	50	Resident physicians at a university internal medicine program	Colorectal	Pt demographics and health	High
Ruff 2005^41^	USA	Determine what risk factors influence the decision of PCPs to screening patients for PCa	Cross-sectional	175	Veteran affairs PCPs (physicians and nurse practitioners)	Prostate	Pt demographics and health Pt psycho-social	High
Sharp 2005^42^	USA	Determine current practices/concerns regarding mammography screening for women of different ages	Cross-sectional, self-reported recommendation	96	Community providers (physicians, physician assistants, nurse practitioners)	Breast	Pt health Pt and clinician psycho-social Health system	High
Sifri 2019^43^	Philadelphia, USA	Assess current PCP approaches to BCa and CRC screening for patients aged 75 + years	Cross-sectional, vignettes	51	Attending physicians, fellows, residents, and nurse practitioners from academic departments at Thomas Jefferson University	Breast	Pt demographics and health Pt psycho-social	High

*PCP* primary care practitioner, *Pt* patient, *CRC* colorectal cancer, *PCa* prostate cancer; *PSA* prostate-specific antigen; *BCa* breast cancer; *USPSTF* United States Preventive Services Taskforce

Eight quantitative studies examined factors associated with recommendations to screen in vignettes.^21–23,29,34,35,40,43^ Six quantitative studies assessed factors associated with self-reported recommendations to screen that were inappropriate (according to guidelines^19,26,28,32,36,37^ or terminal illness^15^). Another quantitative study assessed factors associated with recommending colorectal screening or seeking patient input first.^24^ Other quantitative studies examined factors associated with actual breast screening above 75 years using provider-level data,^18^ PCP views on what factors should be considered when ordering and stopping PSA screening,^38,41^ and concerns around recommending breast screening for women ≥ 75 years (promoting uptake).^42^ Three studies qualitatively explored factors PCPs consider in screening decisions for older adults (≥ 75 years^25,30^, ≥ 80 years^39^; one used vignettes^25^). Five qualitative studies examined decision-making about discontinuing screening or inappropriate screening^16,17,20,27,33^ and one assessed colorectal screening test options.^31^ One study examined PCP views on the extension of national breast screening programs to older women using mixed-methods.^14^

Nine of 20 cross-sectional studies had low risk of bias,^15,19,21–24,26,28,29^ one had low-moderate risk,^32^ six had moderate risk^18,34–38^ and four had high risk^40–43^ (Supplementary Table II). Concerns were sampling (11/20 studies), inadequate sample sizes (14/20 studies), unclear degree of coverage of identified sample (18/20 studies), and measure validity (13/20 studies). Five of nine qualitative studies had low risk of bias,^16,17,20,25,27^ three had low-moderate risk,^30,31,33^ and one had moderate risk.^39^ The mixed-methods study had moderate-high risk of bias (assessed using cross-sectional and qualitative checklist).^14^ Concerns included no philosophical perspective statement (6/9 studies) and limited acknowledgement of the researcher’s location culturally or theoretically (7/9 studies), and their influence on the research (9/9 studies).

**Figure 2:**
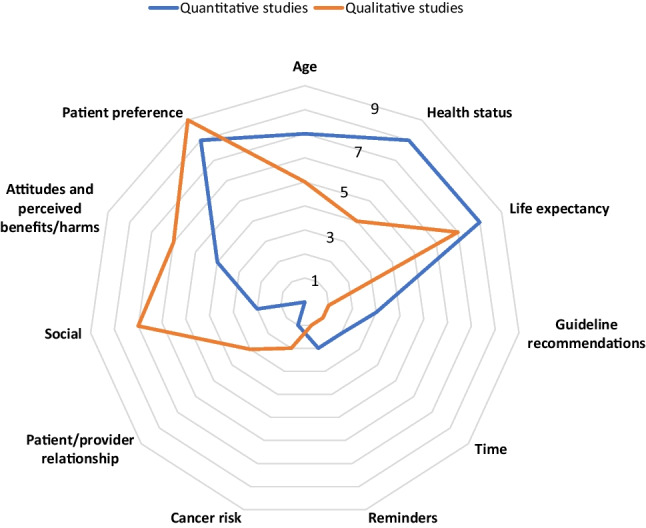
Individual studies reporting influence of factors on PCPs’ recommendations about cancer screening for older adults; further displacement from the center indicates a greater number of individual studies reporting factor as influential; range = 0–9; factors were only included if reported by ≥ 3 studies (across qualitative and quantitative).

Findings from the included studies are summarized in Figure 2. It highlights the factors reported as influential by three or more quantitative or qualitative studies. Furthermore, findings are narratively synthesized below according to patient demographic and health characteristics, patient psycho-social factors, clinician characteristics, clinician psycho-social factors, and health system factors, with the most commonly reported factor summarized first. Influential factors are only summarized in-text if reported by two or more studies. However, all the findings from the quantitative studies (including effect sizes where available) are shown in Table 2, and for the qualitative studies in Table 3 (quotes in Supplementary Table III) and interactions between factors in Table 4.

**Table 2:** Summary of Quantitative Studies Examining Factors Influencing Primary Care Practitioners’ Cancer Screening Recommendations

Study (author, year)	Outcome and analysis details	Variable	Results Means/proportions (%), test statistic (odds ratios [OR], relative risks [RR]), 95% confidence intervals (CIs; unless otherwise specified), *p*-values
Patient demographic characteristics			
Heflin 2006^22^	Intention to offer BCa and cervical screening; vignettes; multi-variable regression	Age	Controlled for age in multi-variable analysis (no significant interaction) Women in frail health: 60% intended to offer BCa screening to the 80-year-old and 31% intended to offer BCa screening to the 90-year-old 34% intended to offer cervical screening to 80-year-old and 13% intended to offer cervical screening to 90-year-old
Kahi 2009^34^	Recommending CRC screening; vignettes; generalized estimation equations	Age	95% would screen a healthy 75-year-old with no comorbidity, versus 66% for healthy 80-year-old and 39% for a healthy 85-year-old
Kistler 2018^35^	Recommending CRC and PCa screening; vignettes and attitudes; generalized linear regression	Age	Recommend screening more frequently for younger than for older patients (70-year-old vs. 90-year-old; *p* < 0.001)
Konety 2006^37^	Self-reported PCa screening recommendation (75 + years); logistic regression	Age	65–70 years: 0.6% of family medicine practitioners and 2% of urologists 71–75 years: 9.3% of family medicine practitioners, 16% of internists, 10% of oncologists, and 14.5% of urologists 76–80 years: 21% of family medicine practitioners, 27.5% of internists, 38% of oncologists, and 41.6% of urologists 80 + years: 64.7% of family medicine practitioners, 52.8% of internists, 48% of oncologists, and 39.5% of urologists Preferred not to stop: 4% of family medicine practitioners, 3.4% of internists, 3.4% of oncologists, and 2% of urologists
Pollack 2012^38^	Attitudes and beliefs about PCa screening; descriptive	Age	32.5% did not have an age at which they typically stop recommending PSA screening For 67.5% who discontinued screening based on patient age, 26.8% at 70 years, 52.4% at 75 years, and 20.7% at > 80 years
Ruff 2005^41^	Self-reported tendency to screen for PCa; descriptive	Age	49 (28.0%) and 67 (38.3%) providers thought age of > 75 years and 80 years respectively substantially decreased their tendency to screen for PCa
		Race	African American race somewhat or significantly increased tendency for screening for 148 (84.6%) practitioners
Walters 2011^14^	Factors influencing BCa screening recommendations (descriptive) and discrete choice experiment (logistic regression)	Age	Screening should only be offered to age range where it was cost effective (63%, 87/138). Patient age had greatest influence in discrete choice experiment. Clinicians 17 × more likely to not screen (compared to undecided) woman > 85 vs < 70 (*p* < 0.001)
Patient health			
Haggstrom 2013^21^	Recommending CRC screening; vignettes; logistic regression	Comorbidity	Physicians were less likely to recommend any screening among patients with unresectable non-small cell lung cancer (34%) than among those who were healthy (96% vs. 34%, *p* < 0.001) or had congestive heart failure (89% vs. 34%, *p* < 0.001), across all age groups
Heflin 2006^22^	Intention to offer BCa and cervical screening; vignettes; multi-variable regression	Health status	Breast: less likely to offer screening if moderately ill (OR = 0.66, 95% CI = 0.51–0.85) or frail (OR = 0.42, 95% CI = 0.33–0.55) compared to healthy woman Cervical: less likely to offer a screening Pap smear to a woman who was either moderately ill (OR = 0.62, 95% CI ¼ 0.47–0.80) or in frail health (OR = 0.32, 95% CI = 0.24–0.43) compared to a woman in good health
		Screening history	Not significantly associated with physicians’ intentions to offer mammography History of normal Pap smears associated with a lower likelihood of offering cervical cancer screening (OR = 0.66, 95% CI = 0.54–0.81)
Kadaoui 2012^23^	Prescribing BCa screening for women 70 + years with <5-year life expectancy	Life expectancy	> 5-year life expectancy: 50% prescribed screening mammography < 5-year life expectancy: 8.6% prescribed screening mammography
Kahi 2009^34^	Recommending CRC screening; vignettes; generalized estimation equations	Comorbidity	75-year-old with no medical problems: 95% would screen; moderate congestive heart failure: 61% screen; moderate chronic obstructive pulmonary disease: 63% screen; severe congestive heart failure: 15% screen; severe chronic obstructive pulmonary disease: 17% screen
		Life expectancy	< 5 years life expectancy (regardless of age): unlikely to screen (9% for 75-year-old, 7% for 80-year-old, 4% for 85-year-old) 75-year-old with < 10 years life expectancy: 43% were likely to screen
		Screening history	75-year-old who had undergone sigmoidoscopy or colonoscopy within preceding 5 or 10 years: most would recommend screening
Lewis 2013^24^	Recommending CRC screening or seeking patient input; vignettes; logistic regression	Health	Good health: 47% would screen, 8% against screening, 45% reported it would depend upon patient input Fair health: 20% would screen, 31% against screening, 49% indicated it would depend upon patient input Poor health: 8% would screen, 66% against screening, 26% would seek patient input first; *p* < 0.0001 across vignettes
		Life expectancy	> 10 years: generally favored screening 2–5 or 6–10 years: more likely to seek patient input prior to making recommendation < 2 years: generally against screening; *p* < 0.0001 across vignettes
Lewis 2008^40^	CRC screening recommendation; vignettes of 75-year-old; logistic regression	Health state	Good health: residents recommended screening (*n* = 33, 66%) or that the patient decide (*n* = 17, 34%) Fair health: 68% would let the patient decide, 26% would recommend screening and 6% would recommend against screening Poor health: 58% would let patient decide, 11% would recommend against screening and 9% would not offer screening at all *p* < 0.0001 across vignettes
		Life expectancy	Resident physicians’ screening recommendations varied with their life expectancy estimates Estimated < 2 years: none recommended screening Estimated > 10 years: recommended that screening be performed (62%) or would let the patient decide (38%) Estimated 2 to < 5 years: 54% recommended that the patient decide Estimated 5–10 years: 63% recommended that the patient decide
Pollack 2012^38^	Attitudes and beliefs about PCa screening; descriptive	Life expectancy	76.8% agreed/strongly agreed that they typically took life expectancy into account when ordering PSA screening 66.4% described it as very difficult or somewhat difficult to estimate life expectancy in clinical practice 69.1% believed it would be helpful to have a clinical decision support tool that would help predict mortality
Ruff 2005^41^	Self-reported tendency to screen for PCa; descriptive	Family history	74.3% were somewhat or significantly more likely to screen
		History of smoking	43.4% were somewhat or significantly more likely to screen
		Vasectomy	28.6% were somewhat or significantly more likely to screen
		Agent Orange exposure	20.6% were significantly more likely to screen
		Life expectancy	< 5 years: biggest deterrent to screening, but only 74 (42.3%) thought this somewhat or significantly decreased their tendency toward screening
Sifri 2019^43^	CRC recommendation; survey, vignettes; descriptive	Life expectancy	84.3% ranked as most important factor determining whether to order cancer screening for someone age > 75
		Medical conditions	70.6% ranked as third most important factor determining whether to order cancer screening for someone age > 75
Sharp 2005^42^	Concerns about BCa screening in women 75 + years; descriptive	Comorbidity	35% mentioned comorbidity as a concern about recommending BCa screening for women older than 75 years
		Functional status	49% mentioned functional status as a concern about recommending BCa screening for women older than 75 years
Walters 2011^14^	BCa recommendations (descriptive) and discrete choice experiment (logistic regression)	Health	68% support that screening should be offered to any women over 50 if she was healthy enough to benefit 36.7% would not screen very healthy, older woman (80–84 years, no comorbidity, raised BCa risk, fully independent and normal cognitive function)
		Comorbidity	Age alone was not rated as highly as comorbidity when considering whether to screen
		Life expectancy	82% rated life expectancy as very important/important
Patient psycho-social			
Boone 2018^19^	Reasons for continuing cervical screening in older women (65 + years)	Patient pressure	45.6% (45.6–51.2%) reported patient sees little downside and wants reassurance
		Patient concern	43.8% (41.1–46.6%) reported patient concern that stopping screening will lead to delayed diagnosis
		Patient choice	38.3% (35.6–41.0%) reported that decision to stop is patient’s choice
		Patient reluctance	36.6% (33.9–39.3%) reported patients would be reluctant to stop when testing has been advocated for decades
		Screen for other cancers	16.9% (14.9–19.1%) reported patients believe screening will detect other types of GYN cancers
		Patient confusion	14.8% (12.9–16.9%) reported patients would be confused as to the purpose of a pelvic examination if cervical screening isn’t performed 14.1% (12.2–16.1%) reported patients confused as to why screening stops when same aged peers continue to receive testing from other providers
		Fear of PCP regret	11.5% (9.8–13.4%) reported patients would fear doctors will change mind in future and conclude screening should not have stopped
Kistler 2018^35^	Recommending CRC and PCa screening; vignettes and attitudes; generalized linear regression	Patient request	Screening recommended more frequently for those who requested it than for those who did not (*p* < 0.001) 52% reported they would recommend PCa screening for a hypothetical 70-year-old patient who requested it
Konety 2006^37^	Self-reported PCa screening recommendation (75 + years); logistic regression	Patient request	75% would consent to patient’s request for PCa screening even if ≥ 75 years old
Lewis 2013^24^	Recommending CRC screening or seeking patient input; vignettes; logistic regression	Patient input	For those who would seek patient input, almost all would make a specific recommendation after obtaining patient input rather than let the patient decide entirely on their own (95% for the good health vignette, 98% for the fair health vignette, and 94% for the poor health vignette)
Pollack 2012^38^	Attitudes and beliefs about PCa screening; descriptive	Patient expectation	74.4% agreed reason for not discontinuing screening was, “My patients expect me to continue getting yearly PSA tests”
Ruff 2005^41^	Self-reported tendency to screen for PCa; descriptive	Patient anxiety	71.2% somewhat or significantly increased tendency to screen for PCa
Sharp 2005^42^	Concerns about BCa screening in women 75 + years; descriptive	Patient resistance	33% mentioned patient resistance as a concern about recommending BCa screening for women older than 75 years
Sifri 2019^43^	CRC recommendation; survey, vignettes; descriptive	Patient preference	82.4% reported patient preference as second most important factor determining whether to order screening for someone age > 75
Clinician characteristics			
Haggstrom 2013^21^	Recommending CRC screening; vignettes; logistic regression	Gender	Females more likely than men to recommend screening for 80-year-old with non-small cell lung cancer
		Specialty	Obstetrician-Gynecologists more likely to recommend screening for 80-year-old with non-small cell lung cancer while physicians who were board-certified or had a full electronic medical record (EMR) were less likely to screen
Heflin 2006^22^	Intention to offer BCa and cervical screening; vignettes; multi-variable analysis	Gender	Breast: females more likely than men to offer a woman with limited life expectancy a screening mammogram (OR = 1.75, 95% CI = 1.21–2.51) Males more likely to underscreen (OR = 2.00, 95% CI = 1.06–3.80) Cervical: No differences existed with respect to gender, specialty, year of graduation, region of the country, or practice type
		Board-certified	Breast: Nonboard-certified physicians were more likely than their certified colleagues to underscreen (22.9% vs 12.3%, *p* = .0061). Nonboard-certified physicians were more likely to underscreen after controlling for other characteristics (OR = 1.86, 95% CI = 1.09–3.18) Cervical: board certification status was associated with a lower likelihood of offering a woman with limited life expectancy a Pap smear (OR ¼ 0.57, 95% CI ¼ 0.36–0.90). No significant factors associated with this underscreening practice pattern
Kadaoui 2012^23^	Prescribing BCa screening for women 70 + years with <5-year life expectancy	Type of practice	Spending time participating in nonclinical activities (no further details provided) was determinant of inappropriate prescription of BCa screening: OR = 1.117 (1.032–1.260)
Kahi 2009^34^	Recommending CRC screening; vignettes; generalized estimation equations	Age	Older providers were less likely than younger providers to recommend screening for an 85-year-old patient who had a clearing colonoscopy within the previous 10 years (*p* = 0.04)
		Graduation location	No main effects
		Board certification	No main effects
		Gender	No main effects
Konety 2009^36^	Self-reported PCa screening recommendation (75 + years); logistic regression	Age	30–40: OR = 1 (ref); 41–50: OR = 1.27, 95% CI = 1.06–1.53; 51–60: OR = 1.62, 95% CI = 1.11–2.35; > 60: OR = 2.06, 95% CI = 1.18–3.60; *p* = .0154
		Degree	Dr of medicine: OR = 1 (ref); Dr of osteopathic medicine: OR = 1.72, 95% CI = 1.07–2.77; Physician assistant-certified: OR = 1.59, 95% CI = 0.91–2.77; *p* = 0.326
		Practice specialty	Family medicine: OR = 1 (ref) Internal medicine, geriatrics: OR = 0.80, 95% CI = 0.51–1.28; Urology: OR = 0.49, 95% CI = 0.23–1.03; Medical oncology, radiation oncology: OR = 0.28, 95% CI = 0.10–0.82; Other 0.12 (0.03–0.54); *p* = .0016
Konety 2006^37^	Self-reported PCa screening recommendation (75 + years); logistic regression	Age	Older providers (> 51 years) were significantly more likely to screen even in elderly men (> 75 years)
		Specialty	Family medicine practitioners were also more likely to screen elderly patients compared with other types of providers
Leach 2012^15^	Over-recommending BCa screening; logistic regression	Specialty	Healthy women 80 years: 70% of FP/GPs and internists would recommend BCa screening versus 86.3% of OB/GYNs. Similarly, more OB/ GYNs (67.5%) than FP/GPs (48.4%) or internists (40.1%) would recommend BCa screening to a woman aged 80 years who had a moderate comorbidity. Compared with FP/GPs (48%), OB/GYNs (67%) had 1.69 times higher odds and internists (33%) had 0.45 times lower odds of over-recommending screening
		Sex	PCPs who were women were 1.40 times more likely than PCPs who were men to over-recommend screening (52%. vs. 43%)
		Race/ethnicty	Being of a race/ethnicity other than non-Hispanic white was associated with a 1.72 times higher odds of over-recommending screening (56% vs. 41%)
		Practice size	PCPs in larger practices were less likely to over-recommend than PCPs in solo practice (2–5 PCPs: OR, 0.66; 6 PCPs: OR, 0.71) (43% vs. 53%)
		Practice location	Practice location was marginally significant predictor of over-recommending screening (OR, 1.47; *p* = .0517) (48% urban vs. 38% rural)
Yasmeen 2012^29^	Decision to recommend BCa screening; vignette; logistic regression	Physician type	Obstetrician-gynecologists (OBGYN, 37%) more likely to recommend BCa screening life expectancy < 5 years compared to internal medicine (IM, 17%) and family medicine (FM) doctors (16%) 86% OBGYN (86%) indicated “always” recommending BCa screening to BCa screening women (70–89 years), compared to 67% for IM and 59% for FM (*p* ≤ .001 for both comparisons). OBGYN more likely than FM to recommend mammography for women 70 + (OR 4.5, 95% CI: 2.6–7.4)
Walters 2011^14^	Factors influencing BCa screening recommendations (descriptive) and discrete choice experiment (logistic regression)	Gender	Females were half as likely as males to choose not to screen compared to being undecided (female vs. male clinician gender, relative risk for not screening vs. undecided 0.52, 95% CI 0.32–0.84, *p* = 0.008)
Clinician psycho-social			
Boone 2018^19^	Reasons for continuing cervical screening in older women (65 + years)	Fear of missing cancer	56.6% (53.8–59.3%) believe stopping could miss preventable cancer
		Keep patient coming	35.9% (33.2–38.6%) continues to maintain relevance of yearly preventive visit
		Difficult to explain	26.9% (24.5–29.5%) felt that trying to convince a patient that not screening is somehow of individual benefit is difficult to convey
		Fear patient thinking not complete visit	17.8% (15.7–20.0%) fears perception by patients that an examination without screening is a less complete preventive visit
		Fear patient thinking it is cost related	12.2% (10.4–14.1%) fears stopping will be perceived by patients as primarily to benefit payers, not patients
Haas 2017^18^	Whether woman received BCa screening 2 years after PCP visit; logistic regression	Uncertainty	Use of BCa screening was highest among patients cared for by PCPs who were uncertain about BCa screening effectiveness (50.7%). Differences in use by beliefs about effectiveness were significant (*p* = 0.003) after controlling for patient characteristics. There was no association between PCP beliefs about effectiveness and receipt of annual screening for this age group
Kadaoui 2012^23^	Prescribing BCa screening for women 70 + years with > <5-year life expectancy	Attitudes	Favorable attitude to screening was determinant of inappropriate prescription of BCa screening OR = 4.279 (2.534–7.623)
		Screening skills	Lower screening skills were determinant of inappropriate prescription of BCa screening: OR = 0.783 (0.624–0.974)
Kistler 2018^35^	Recommending CRC and PCa screening; vignettes and attitudes; generalized linear regression	Attitudes	More positive PCa screening attitudes increased likelihood of recommending PCa screening for 90-year-old by 30% (RR ratio 1.3, 95% CI 1.1–1.5) More positive CRC screening attitudes increased likelihood of recommending CRC screening for 90-year-old by 30% (RR ratio 1.3, 95% CI 1.2–1.5)
Lewis 2008^40^	CRC screening recommendation; vignettes of 75-year-old; logistic regression	Uncertainty	PCPs who reported uncertainty about the likelihood of patient benefiting from screening were more likely to recommend that the patient decide about screening across all vignettes at age 75 years rather than providing recommendation (OR = 3.26, 95% CI = 1.27–8.38; RR = 1.98; 95% CI = 1.18–5.99)
Pollack 2018^32^	Self-reported BCa screening recommendation; logistic regression	Anecdotal experiences	Reporting 1 + social network member with a poor prognosis who was not diagnosed by screening increased likelihood of recommending BCa screening to women 75 + years compared with physicians who did not report a social network member in this category (84.0% vs 68.3%, *p* < .001)
Radhakrishnan 2018^26^	Self-reported BCa screening recommendation; logistic regression	Potential regret (scale)	Increasing levels of potential regret (1 point on 5-point scale) increased likelihood of recommending BCa screening to older women (OR = 4.62; 95% CI: 3.50–6.11). Items: liability from not ordering screening, fear of missing lethal cancers, patient expectations to receive screening and difficulty reconciling clinical uncertainty with varying organizational guidelines
		Concern for patient-related hazards (scale)	Increasing concern for patient-related hazards decreased odds of recommending BCa screening to older women (OR = 0.68; 95% CI = 0.56–0.83). Items: concern patients will need additional tests and do not end up having cancer, worry about finding cancer that would never have caused problems
Sharp 2005^42^	Concerns about BCa screening in women 75 + years; descriptive	Provider assessment of value of test	43% mentioned provider assessment of value of the test for women this age as a concern about recommending BCa screening for women older than 75 years
Health system			
Boone 2018^19^	Predominant reasons for continuing cervical screening in older women (65 + years)	Time	55.3% (52.5–58.1%) reported that it takes less time to perform screening than explain why not 45.9% (43.1–48.7%) reported frustration over inadequate time to address confusion why screening no longer required
		Access to records	37.3% (34.7–40.2%) reported that difficulty obtaining prior records causes concern that patient may not be truly low risk and appropriate to stop
Kistler 2018^35^	Recommending CRC and PCa screening; vignettes and attitudes; generalized linear regression	Guidelines	90% reported that USPSTF guidelines were very or extremely influential in CRC and PCa screening recommendations
		Clinical reminders/ performance measures	Clinical reminders/performance (PCa: 2.6, CRC: 2.9; 4-point scale; higher = more influence) measures influenced recommendations for 70-year-old male more than short patient visits (PCa: 2.1, CRC: 2.0) or lawsuit worries (PCa: 2.3, CRC: 2.1)* (both *p* < 0.001)
Leach 2012^15^	Over-recommending BCa screening; logistic regression	Guidelines	Rating the American Congress of Obstetricians and Gynecologists guidelines as “very influential” (54%, OR 1.37; *p* = .0512) compared with “somewhat influential,” “not influential,” or “not applicable/ not familiar with” (38%) was a marginally significant predictor of over-recommending screening
Pollack 2012^38^	Attitudes and beliefs about PCa screening; descriptive	Time	66.4% agreed reason for not discontinuing screening: “It takes more time to explain why I’m not screening than to just continue screening”
Siembida 2017^28^	Self-reported BCa screening recommendation; logistic regression	Receiving reminders	Those who received reminders were more likely to report recommending BCa screening to women aged > 75 years (84%; 94% CI: 77–90% compared to those who did not (65%; 95% CI: 62–69%)
Sharp 2005^42^	Concerns about BCa screening in women 75 + years; descriptive	Inconsistent guidelines	33% mentioned inconsistent guidelines as a concern about recommending BCa screening for women older than 75 years
		Cost to patient	20% mentioned cost to patient as a concern about recommending BCa screening for women older than 75 years
Sifri 2019^43^	CRC recommendation; survey, vignettes; descriptive	Decision support tools	60.8% reported they would find methods for estimating life expectancy helpful 64.7% reported they would find decision aids for providers to estimate benefits/harms helpful 66.7% reported they would find decision aids for patients that present personalized benefits/harms of cancer screening helpful
Yasmeen 2012^29^	Decision to recommend BCa screening; vignette; logistic regression	Guidelines	45% of respondents rated the USPSTF guidelines as “extremely influential”

*PCP* primary care practitioner, *HCP* healthcare professional, *CRC* colorectal cancer, *PCa* prostate cancer, *PSA* prostate-specific antigen, *BCa* breast cancer, *USPSTF* United States Preventive Services Taskforce

**Table 3:** Summary of Qualitative Studies Examining Factors Influencing Primary Care Practitioners’ Cancer Screening Recommendations

Study (author, year)	Outcome details	Results
Patient demographic and health characteristics		
Austin 2021^17^	Experiences discussing BCa screening with older patients, influence of guidelines, overscreening perceptions	*Life expectancy*: a few described how it is challenging to know which women are less likely to benefit from BCa screening and, despite feeling life expectancy was pertinent, they did not feel comfortable discussing it with their patients
Enns 2021^20^	BCa, PCa and CRC decisions in general and in those with limited life expectancy	*Age*: Some used the same age cutoff for stopping across all three types. Others had different ages for stopping for each cancer
Lewis 2009^25^	CRC screening decisions using two vignettes of 78-year-old women in fair and poor health	*Age*: no quote available
		*Living situation of the patient:* no quote available
		*Previous screening behaviour:* no quote available
		*Life expectancy*: PCPs felt the potential benefits of screening were delayed and that life expectancy of 5–10 years was necessary to reap benefits. But there was underlying uncertainty
Oshima 2021^30^	BCa screening practice patterns with older women including discussions about stopping	*Health status:* all PCPs reported that a patient’s health status affected how they approached recommendations
		*Life expectancy:* Some (*n* = 5) assessed health status and 10-year life expectancy by considering comorbid conditions (e.g., renal failure, heart failure, coronary artery disease, metastatic cancer, neurologic degenerative processes). Several (*n* = 4) were uncertain in accurately estimating prognosis
		*Functional status:* used to decide whether or not to discuss screening
		*Risk of breast cancer:* PCPs were more likely to bring up BCa screening if there was personal/family history, or other relevant risk factors, e.g., obesity
Park 2021^31^	PCP perceptions of CRC screening test options in older adults	*Age*: PCPs reported resorting to stool tests in older patients who would be too high risk for a colonoscopy
		*Functional status*: PCPs considered burdens of getting to the screening facility (e.g., mobility and/or transportation challenges)
		*Life expectancy*: some stopped all screening in patients with clearly limited life expectancy. Others recommended stool tests for patients with limited life expectancies due to low upfront risk (especially if patient requested)
		*Comorbidities*: some commented on using stool tests in patients with comorbidities that would make colonoscopy too risky or in patients who are on blood thinners
Schoenborn 2020a^27^	Decisions for 2–3 patients per PCP: ≥1 who had screened in previous year and 1 who had not	*Age:* no quote available
		*Functional status, ability to tolerate further tests or treatment*, *quality of life*: no quotes available
		*Baseline risk of cancer, family history of cancer:* no quote available
		*Health status:* decisions not limited to options of continuing vs stopping. Rather than stopping altogether in patients with significant health concerns, PCPs considered other alternatives (e.g., less frequent screening, relying on physical exam (e.g., breast and prostate exams), less invasive tests, or deferring the decision to a later time)
Schoenborn 2020b^33^	Using life expectancy to decide when screening should stop	*Life expectancy*: some agreed basing decision to stop on limited life expectancy but others disagreed. Many used < 10 years as threshold
		*Distrust of life expectancy predictions*: clinicians were skeptical of prediction tools and felt the existing models may miss important variables (e.g., family history of longevity), and did not account for changes in patient status or medical technology
		*Using life expectancy felt impersonal*: Some felt uncomfortable even when they understood the rationale for the guidelines
		*Concern for bias in considering life expectancy*: bias in terms of race and cost are introduced when using life expectancy and developing prediction tools
Schonberg 2006^39^	Explore decision-making about BCa screening in women aged ≥ 80 years	*Age:* three commented that they were less likely to recommend screening to patients as they aged into their late 80 s or 90 s
		*Availability of further tests or treatment*: eight noted that the availability of acceptable treatments for elderly women with BCa influenced their screening recommendations
		*Life expectancy:* eight described recommending screening to patients they perceived to have adequate life expectancy and not discussing screening or discussing stopping with patients they perceived to have very limited life expectancy
Walters 2011^14^	Views on extending BCa screening to women ≥ 70 years	*Life expectancy and fitness*: the effect of comorbidity, frailty and cognitive decline were all viewed as important if healthcare professionals were considering whether to recommend continuing screening. It was generally felt that a woman should continue to be screened if she was fit enough to benefit
		*Progressive increase in breast cancer risk with older age*
		*Cognitive impairment*: most would not recommend screening for older women with significant cognitive impairment who would be unable to give informed consent should they need treatment, due to the marginal benefits
Patient psycho-social		
Austin 2021^17^	Experiences discussing BCa screening with older patients, influence of guidelines, overscreening perceptions	*Patient autonomy*: several reported that an in-person visit was not required for older women to obtain a referral for BCa screening and that they could call the provider’s office and speak with a nurse who can generate an order or have the referral signed off by another provider who is unfamiliar with the patient’s history
Lewis 2009^25^	CRC screening decisions using two vignettes of 78-year-old women in fair and poor health	*Patient personality:* whether a patient had previously been aggressive about screening or were proactive about screening
		*Patient-provider relationship:* no quote available
		*Family support:* Another aspect was opinions of family members about screening
		*Patient autonomy:* PCPs reported a patient in fair health had a highly autonomous role. Many emphasized their role was to provide information to help the patient understand options
Oshima 2021^30^	BCa screening practice patterns with older women including discussions about stopping	*Patient preference:* all strongly valued a patient’s desire for continued screening as rationale for continuing. While most older women were perceived as eager to stop, PCPs also acknowledged that patients’ personal experiences with screening influenced the degree to which they felt invested in BCa screening
		*Patient satisfaction:* four mentioned their recommendations were influenced by knowing that performance evaluations depend on patient satisfaction, e.g., Press Ganey scores. This led to reluctance to recommend an alternative if a patient had a strong preference for continued screening
		*Patient post-screening preference:* seven emphasized the importance of knowing how invested women were in the outcomes following screening
		*Patient-provider relationship*: strong relationships gave PCPs more insight into patient preferences, increased patient trust of PCP recommendations, and helped PCPs more easily personalize discussions and recommendations
Park 2021^31^	PCP perceptions of CRC screening test options	*Patient choice:* patient preference was a strong influence on the choice of screening tests. Stool tests were considered alternatives when patients refused colonoscopy
Rowe 2021^16^	PCP explanations for overuse of PCa screening in men ≥ 75 years	*Patient preference*: PCPs were aware and knowledgeable of guidelines, but many deferred to patient preference when deciding whether to order a test. Another recognized that PSA screening in older men probably should not be ordered but felt some were still insistent or expectant
Schoenborn 2020a^27^	Decisions for 2–3 patients: at least 1 who had screened in previous year and 1 who had not	*Patient request*: most would give in to the patients’ request for screening even if they otherwise would have stopped screening
		*Anecdotes about family and friends*
Schoenborn 2020b^33^	Using life expectancy to decide when screening should stop	*Patient choice*: PCPs generally felt that patients should be able to make the ultimate decision as long they were informed about the benefits and risks, even if that meant choosing screening when they had limited life expectancy
Schonberg 2006^39^	Explore decision-making about BCa screening in women aged ≥ 80 years	*Patient preference*: if patients had a preference about screening PCPs followed the patient’s preference
		*Patient-provider relationship:* six described that a longstanding doctor-patient relationship and patient trust can facilitate these discussions
Walters 2011^14^	Views on extending BCa screening to women ≥ 70 years	*Patient choice:* “Quite a lot of elderly patients ask if they can come back for screening when it ceases.”
Clinician psycho-social		
Austin 2021^17^	Experiences discussing BCa screening with older patients, influence of guidelines, overscreening perceptions	*Benefits vs. harms*: a few described how other PCPs believe that the benefits of screening outweigh the potential harms of not screening
		*Past experiences:* PCP colleague’s decisions to continue screening indefinitely may be due to past experiences; no quote available
		*Avoid confusion:* PCP colleague’s decisions to continue screening indefinitely may be due to avoid confusion among older women; no quote available
		*Fear of malpractice*: PCP colleague’s decisions to continue screening indefinitely may be due to fear of malpractice
Enns 2021^20^	BCa, PCa and CRC screening decisions in patients with limited life expectancy and in general	*Accuracy and efficacy of test*: some felt more strongly about continuing colonoscopy due to being a more reliable way of finding/preventing advanced cancer
		*Minimising direct harm*: others would forego colonoscopies in a patient while continuing PSA or BCa screening
		*Downstream harms*: clinicians considered downstream harms even when the upfront harm is low such as for PSA test
		*Negative consequences of late-stage cancers*: Others focused on the negative consequences of late-stage cancers when prioritizing different screenings
		*Cancers that are easier to treat*: one PCP would continue BCa screening in a patient while stopping other screenings because cancers detected by mammogram require treatments that are less risky and invasive than those used to treat other cancers
Lewis 2009^25^	CRC screening decisions using two vignettes of 78-year-old women in fair and poor health	*Gestalt process:* it was difficult for PCPs to express how they put all considered factors together to decide whether screening was worthwhile. Some reported that the decision was based on more of gestalt (i.e., a feeling of knowing the patient and their medical problems) and emphasized the importance of a long-term relationship
		*Concern about harm:* the major concern was when there was no chance that a patient could benefit and also about causing distress from colonoscopy
		*Influence of screening test on management:* physicians considered what they would do with information from a screening test and whether it would change their future management of the patient. If they were unlikely to pursue treatment, they were less likely to recommend screening
		*Difficulty weighing many factors:* Some reported that the decision-making process for CRC was difficult due to the sheer number of factors that they had to weigh
Oshima 2021^30^	BCa screening practice patterns with older women including discussions about stopping	*Evidence:* seven felt there was inadequate evidence to suggest a best practice for BCa screening in women ≥ 75, one felt there was enough evidence to recommend against screening in older women. All reported frequently engaging in shared decision-making on whether to pursue routine BCa screening, however this was particularly important among PCPs who felt that current guidelines were unclear
Park 2021^31^	PCP perceptions of CRC screening test options in older adults	*Familiarity with newer tests*
		*Test effectiveness:* some were concerned that stool tests are not as effective as colonoscopy in detecting advanced adenomas
Rowe 2021^16^	PCP explanations for overuse of PCa screening in men ≥ 75 years	*Knowledge*: PCPs were aware of guideline content, suggesting over testing was not due to a knowledge deficit. General knowledge of guidelines was apparent in the responses to discrete questions. (e.g., 67% agreed that harms of PCa screening outweighed benefits for the average 77-year-old man)
		*Underestimation of harms*: few discussed downsides of PCa screening. One mentioned they would, but later discussed not wanting to miss PCa in an older healthy man
		*Resistance to change*: many reported the desire to not change management for patients doing well, preferring to maintain the status quo
Schoenborn 2020a^27^	Decisions for 2–3 patients per PCP: at least 1 who had screened in previous year and 1 who had not	*Anecdotes*: clinicians mentioned various anecdotal experiences involving patients or their personal experiences that were factors in their cancer screening practice
		*Risks from screening test:* no quote available
		*Gestalt process:* for 26/53 patients, their PCP described the decision as not a conscious one or was not able to recall how the decision was made even after medical record review (in patients with and without recent screening). Sometimes after record review, PCPs said they should have made the opposite decision for some
Schoenborn 2020b^33^	Using life expectancy to decide when screening should stop	*Non–mortality-related benefits of screening***:** PCPs mentioned that screening may provide other benefits that make it worthwhile, even if it did not impact the patient’s mortality; including quality of life, less extensive treatment, reassurance, and positive changes triggered by knowing a cancer diagnosis
		*Difficulty of applying population-based screening data to individual patients*
		*Overscreening perceptions:* 18/30 PCPs perceived there was overscreening in older adults. Others did not believe there was or thought it was acceptable. Others disagreed with how overscreening is defined
Schonberg 2006^39^	Explore decision-making about BCa screening in women aged ≥ 80 years	*Not wanting to discuss stopping screening*: 9 PCPs described difficulties when discussing stopping screening with women ≥ 80 years; 6 reported it can be uncomfortable
Walters 2011^14^	Views on extending BCa screening to women ≥ 70 years	*Difficulty explaining risks vs benefits*: several HCPs reported the potential difficulties of explaining the risks vs. benefits of BCa screening to women ≥ 70 years, as well as the controversy regarding the factors to consider in this decision-making process
		*Impact of downsides of screening*: overdiagnosis, false positive results and the impact of overtreatment were felt to be more pressing in the older age group
Health system		
Austin 2021^17^	Experiences discussing BCa screening with older patients, influence of guidelines, overscreening perceptions	*Guideline recommendations*: all stated that they followed the guideline recommendations for BCa screening released by their respective professional organizations, mainly the USPSTF and American College of Obstetrics and Gynecology. However, providers discussed that providers within their own specialty and/or clinic did not always adhere
		*No within-system consensus*: PCPs felt the annual reminder letter from outside their clinic created conflict/confusion around which provider specialty is in charge of BCa screening and made it difficult for them to discuss stopping or reducing screening during an appointment
		*Educational resources* + *electronic health record*: several stated older women and PCPs could receive educational resources about the harms and limited benefits of BCa screening to help facilitate discussions. A couple suggested utilizing the electronic health record to identify women for whom stopping (or reducing frequency of) BCa screening are recommended (e.g., < 10-year life expectancy) and to customize system-generated reminder letters based on individual BCa risk and health status
Oshima 2021^30^	BCa screening practice patterns with older women including discussions about stopping	*USPSTF recommendations*: seven followed USPSTF recommendations for BCa screening in daily practice, beginning discussions at age 40, with an increased emphasis on routine screening every 1–2 years from age 50–75
		*Financial costs*: five did not consider financial costs when discussing BCa screening, due partly to difficulty determining cost given healthcare pricing opacity, but one did
		*Institutional quality metrics:* not reported as consideration in recommendations. Two mentioned that metrics did not apply for patients ≥ 75 years
		*Decision support tools*: six indicated a patient-facing handout outlining the current guidelines surrounding BCa screening along with considerations for patients would be helpful. Two suggested EMR notifications alerting PCPs that a mammogram is due would be helpful. One said reminder alerts with updates on guideline recommendations would be useful, while another mentioned a provider-facing screening decision tool could aid in clinical decision-making
Park 2021^31^	PCP perceptions of CRC screening test options in older adults	*Quality metrics:* getting scored on how well PCPs screen patients
		*Not returning tests:* not screening because people do not bring them back
		*Cost effectiveness*: some were concerned that stool tests are not cost effective since the newer options are almost as expensive as a colonoscopy
Rowe 2021^16^	PCP explanations for overuse of PCa screening in men ≥ 75 years	*Guidelines*: PCPs voiced awareness of clinical recommendations to avoid overuse in older adults. When asking how one might approach PSA testing in an older man, one PCP commented: “I’d just tell him the guidelines and see what his response was.”
Schoenborn 2020a^27^	Decisions for 2–3 patients: at least 1 who had screened in previous year and 1 who had not	*EMR alerts*: associated with less deliberate decisions. Screened patients: often ordered from routine process or triggered by alerts (BCa and CRC screenings for patients < 75). Patients not screened: absence of alerts, screening not being mentioned, reliance on alerts
		*Specialists*: PCPs reported less control over decisions when patients saw a specialist
Schoenborn 2020b^33^	Using life expectancy to decide when screening should stop	*Guidelines limit access:* PCPs were concerned that the guidelines will be implemented in a way that limits patient access and/or undermines patient-centered care
		*Skeptical about guidelines:* anticipated regret when new evidence comes out
Schonberg 2006^39^	Counselling about BCa screening to women aged ≥ 80 years	*Need better evidence and guidelines: n* = 6
		*Time to provide information:* 6 described how difficult it was for patients to understand the risks and 3 reported that this discussion can take a great deal of time
Walters 2011^14^	Views on extending BCa screening to women ≥ 70 years	*Equality of access:* current system viewed as favoring higher socio-economic groups
		*Cost effectiveness:* several strongly believed screening should fully cease when there were marginal benefits to the woman (i.e., not cost effective or efficient and “overburdened” the service)
		*Burden on health system:* in a system that was currently struggling to cope, several argued it was not a political imperative to promote availability of BCa screening to older women for fear of being “over burdened.”
		*Difficulty of selective screening:* having GPs selectively advise screening for older women raised concerns regarding time, capacity and workload issues. Without selectivity, concerns were for harms done to less fit women. With selectivity some commented that this just introduced an additional, unnecessary barrier to access. Although HCPs were aware that there were currently several problems with the current system of voluntary self-referral (access and uptake), there was no consensus about how the system could be improved

*EMR* electronic medical record, *PCP* primary care practitioner, *HCP* healthcare professional, *CRC* colorectal cancer, *PCa* prostate cancer, *PSA* prostate-specific antigen, *BCa* breast cancer; *USPSTF* United States Preventive Services Taskforce

See Supplementary Table III for quotes, except where stated that no quote is available

**Table 4:** Summary of Studies That Examined Interactions Between Factors Influencing Primary Care Practitioners’ Cancer Screening Recommendations

Study and year	Outcomes and analysis	Factors	Results Means/proportions (%), test statistic (odds ratio [OR]), 95% confidence intervals (CIs; unless otherwise specified), *p*-values
Haggstrom 2013^21^	CRC vignettes; Proportion recommending screening	Age x comorbidities	Age 65: 100% recommend screening if the patient was healthy and 97% recommended screening if patient had congestive heart failure Age 80: 90% recommend screening if patient was healthy and 71% recommended screening if patient had congestive heart failure (*p* < 0.001)
		Age x comorbidities x screening test type	Healthy: physicians less often recommended FOBT alone than colonoscopy (with or without FOBT) for 65-year-olds (2% vs. 91%), or 80-year-olds (19% vs. 73%), with a chi-square *p* value of < 0.001 Congestive heart failure: physicians still less often recommended FOBT alone than colonoscopy for 65-year-olds (19% vs. 64%), although physicians more often recommended FOBT alone than colonoscopy for 80-year-olds (54% vs. 32%), *p* < 0.001 Unresectable non-small cell lung cancer: physicians recommended FOBT alone with the same frequency or more often than colonoscopy for 65-year-olds (50% vs. 41%), and 80-year-olds (65% vs. 28%), *p* < 0.001
Kahi 2009^34^	Likelihood of recommending CRC screening — likely vs unlikely	Age x comorbidity, life expectancy and past screening	75-year-old: PCPs more likely to recommend screening in the presence of milder comorbidity, longer life expectancy, and longer time since recent screening. Trends were maintained for 80 and 85-year-olds, except for prior screening history, which was no longer a factor for 85-year-old
		Age x screening history	75-year-old who had undergone clearing colonoscopy within preceding 5 years: 53% likely to screen 80-year-old who had undergone clearing colonoscopy within preceding 5 years: 23% would screen (*p* < 0.0001)
Kistler 2018^35^	Recommending CRC and PCa screening; vignettes and attitudes; generalized linear regression	Age x patient request	70-year-old: more likely to recommend screening with patient request compared to no patient request (52% vs. 28%) 90-year-old: no difference with patient request (7% vs. 2%, PCa) (interaction approached significance, *p* = 0.08)
		Age x screening test type	CRC: 70 years — 95% vs. 90 years — 4%; PCa: 70 years — 39% vs. 90 years — 3%; *p* < 0.001 for interaction
		Patient request x screening test type	PCa screening for 90-year-old with patient request: 7% recommended screening CRC screening for 90-year-old with patient request: 10% recommended screening
Lewis 2013^24^	CRC screening decisions; two vignettes of 78-year-old women in fair and poor health	Age x life expectancy x comorbidities	“…you could be 75, but you can have 0 life expectancy because of co-morbidities or you could be 85 and have a 10-year life expectancy.”
		Life expectancy x functional status x health status	Good health: PCPs use average life expectancies of persons at that age in their decisions. Poor health: severity of the health conditions and functional status became the focus
		Patient preference x health status	Fair health: shared decision-making approach. “I would always, at least, talk about it and offer it if their life expectancy could be 10 years. The first question I ask is “are you interested in doing this?”” Poor health: some would bring up screening, but others would only discuss it if the patient asked. “Bringing it up is the reasonable thing, but then you have to be very, very honest with them and you have to say look it here’s a situation what are you going to really do if they tell you there’s there and we have to do something about it? Do you really want to know that?” OR would be thinking about other issues “[I] Wouldn’t even think about it.”
Pollack 2012^38^	Multi-variable logistic regression of barriers to discontinuing PCa screening	Age x life expectancy	Those who they had an age at which they typically discontinued screening also were significantly more likely to take life expectancy into account (59.3% considers age and life expectancy, 8.1% considered age not life expectancy, 20.3% considered life expectancy not age, 12% did not consider either)
		Life expectancy x difficulty estimating life expectancy	Those who had difficulty estimating life expectancy were not less likely to take life expectancy into account compared with those who did not report difficulty
		Patient expectation x PCP race	Black providers less likely than non-black providers to agree with “My patients expect me to continue getting yearly PSA tests” (OR 0.25, 95% CI 0.07–0.92)
		Uncertainty x PCP race	Black providers less likely than non-black providers to agree with “I am uncomfortable with the uncertainty if I discontinue screening” (OR 0.16, 95% CI 0.03–0.81)
		Time x PCP race	Black providers less likely than non-black providers to agree with “It takes more time to explain why I’m not screening than to just continue screening” (OR 0.18, 95% CI 0.05–0.72)
Ruff 2005^41^	Self-reported tendency to screen for PCa; descriptive	Patient anxiety x PCP gender	Patient anxiety about PCa was more likely to prompt female providers to screen (*p* = 0.013)
Siembida 2017^28^	Multi-variable logistic regression	Guidelines x reminders	Association between receiving reminders and recommending screening for women > 75 years did not differ depending on the guidelines physicians trusted (*p* = .26)
Yasmeen 2012^29^	Descriptive	Guidelines x specialty	FP (53%) and IM (47%) were more likely to find USPSTF guidelines extremely influential than OBG (25%) (*p* ≤ .001 for both comparisons) OBG were more likely to describe American Cancer Society (*p* = 0.07) and American College of Obstetricians and Gynaecologists (*p* ≤ 0.001) guidelines very influential on their practice compared to FP and IM

*PCP* primary care practitioner, *CRC* colorectal cancer, *PCa* prostate cancer, *BCa* breast cancer, *FOBT* fecal-occult blood testing, *USPSTF* United States Preventive Services Taskforce

### Patient Demographic Characteristics

#### Patient Age

Twelve studies (four of good quality) reported that PCPs’ screening recommendations for older adults were influenced by their age.^14,20,22,25,27,31,34,35,37–39,41^ Quantitative studies using vignettes and linear regression models found greater likelihood of recommending screening to 80-year-olds compared to 90-year-olds;^22^ to 75-year-olds compared to 85-year-olds;^34^ and to 70-year-olds compared to 85- or 90-year-olds.^14,35^ However, these studies had moderate^34,35^ to high risk of bias^14^ and the one study with low risk of bias was conducted in 2006.^22^ In descriptive and qualitative studies, PCPs reported using specific age cutoffs in deciding whether to screen or not. For example, 67.5% reported that they discontinued prostate screening based on age^38^ and “I’ll just do 75 for everything.”^20^

### Patient Health Characteristics

#### Life Expectancy

Fifteen studies (four of good quality) found that PCPs considered life expectancy an important factor in screening recommendations, including being less likely to recommend screening for individuals with limited life expectancy as presented in vignettes (< 5 years^23,34,41^ or < 2 years^14,24,40^).^14,17,25,30,31,33,38,39,43^ In qualitative studies, PCPs determined clearly limited life expectancy by considering comorbidities, frailty, and cognitive decline, but also reported difficulty or uncertainty about these estimations^38^ or disagreed with using life expectancy to guide decision-making. ^33,38^ When life expectancy was 5–10 years in vignettes, PCPs were more likely to defer to the patient’s preference for colorectal screening.^24,40^

#### Health Status, Comorbidities and Functional Status

Five quantitative studies, including three of good quality, found that PCPs were less likely to offer screening (or more likely to recommend against^24^) to those in poor health compared to good health^22,40^ or to those with more severe comorbidity.^21,34^ PCPs also described comorbidity as an important factor in decision-making in six studies (two of good quality) ^14,27,31,33,42,43^ and one study of poor quality found 68% felt that breast screening should be offered to healthy women regardless of their age.^14^ Five qualitative studies, including two of good quality, highlighted how PCPs assessed health status globally or based on “gut feeling” (e.g., considering comorbidities, functional status, life expectancy) when making screening recommendations.^14,25,27,30,31^ One quantitative study of lower quality and one qualitative study of good quality also found functional status and ability to tolerate further tests/treatment were influential.^27,42^

#### Cancer Risk

One quantitative study of low quality^41^ and two qualitative studies of moderate quality^30,46^ reported that risk due to family or personal history of cancer influenced PCP recommendations for breast and prostate screening.

#### Screening History

Screening history influenced PCPs in two studies whereby history of normal cervical screening reduced likelihood of offering screening to older women (good quality)^22^ and longer time since last colonoscopy (> 5 years vs < 5 years) increased likelihood of recommending colonoscopy.^34^

### Patient Psycho-social Factors

#### Patient Personal Preference

Eight quantitative studies (2 of good quality) and nine qualitative studies (4 of good quality) found that PCPs considered patient preference (also conceptualized across studies as patient’s request, expectation, autonomy, satisfaction, experiences, or anxiety), as influential in screening recommendations.^14,16,17,19,24,25,27,30,31,33,35,37–39,41–43^

### Clinician Characteristics

#### Influence on Recommendations

In three lower quality studies, clinician age influenced recommendations, such that older PCPs were less likely than younger PCPs to recommend colorectal screening for 85-year-olds^34^ but more likely to recommend prostate screening for men ≥ 75 years.^36,37^ There were mixed findings for the impact of gender and specialty, as two studies (one of good quality) reported no impact for colorectal screening.^22,34^ However, other studies of lower quality found female PCPs in England were twice as likely to be undecided about breast screening compared to males (who were more likely to recommend against)^14^ and family medicine PCPs were more likely to recommend prostate screening for men ≥ 75 years in the USA than medical and radiation oncology specialty providers.^36,37^

#### Influence on Inappropriate Recommendations (Based on < 5-Year Life Expectancy or Moderate-Severe Comorbidity Including Non-small cell Lung Cancer or Ischemic Cardiomyopathy with Dyspnea)

All studies that assessed influence of clinician characteristics on inappropriate screening recommendations were of good quality. Internal medicine and family medicine doctors were less likely to recommend inappropriate colorectal^21^ or breast screening^29^ compared to obstetrics and gynecology physicians. Females were more likely to recommend inappropriate colorectal^21^ or breast screening^22^ than males (but no difference for cervical).^22^

### Clinician Psycho-social Factors

#### Attitudes and Perceived Benefits/Harms

PCPs considered direct and downstream harms for screening recommendations in four studies, including three of good quality (i.e., overdiagnosis, overtreatment, false positives).^20,25,26,33^ However, two qualitative studies (one of good quality) highlighted differences in PCP recognition of screening harms for older adults, whereby some PCPs did not believe overscreening in older adults occurred, felt it was acceptable, or disagreed with how it is defined based on limited life expectancy.^17,33^ Similarly, a small study found that PCPs recommend inappropriate prostate screening despite knowing that harms may outweigh benefits.^16^ PCPs were more likely to recommend screening when concerned about missing cancers in three good quality studies,^19,20,26^ and when the test was perceived as accurate/effective.^20,31^ Uncertainty led to higher breast screening rates and recommendations in two more recent studies (one of good quality)^18,26^ or deferring to patient preference rather than provide a recommendation in one study from 2008.^40^ Positive attitudes towards screening were associated with recommending screening in two studies (one of good quality).^23,35^ PCPs reported decisions about screening based on a gut feeling, not necessarily conscious or deliberate,^27^ given there were so many factors to consider.^25^

#### Social Factors

Three studies (one of good quality) found that PCPs reported inappropriately screening due to difficulty discussing risks and benefits or stopping screening.^14,19,39^ Anecdotal experiences with patients^27^ or their own social networks^32^ also influenced PCP decision-making. Fear of negative patient appraisal and liability concerns led PCPs to continue inappropriate cervical screening^19^ or recommend breast screening to older women.^26^ PCPs also reported patient-PCP relationship as an important factor in three qualitative studies.^25,30,39^

### Health System Factors

#### Guidelines/Recommendations

Four studies (two of good quality) reported that guidelines were influential in PCPs’ screening recommendations including US Preventive Services Task Force (USPSTF),^29,30,35^ American Cancer Society,^30^ and American College of Obstetricians and Gynecologists^15^ guidelines. However, three qualitative studies highlighted negative views towards guidelines, including concerns of inequality,^14^ undermining patient-centered care,^33^ and inadequate evidence to support best practice.^30^ In older studies, PCPs also reported that better guidelines were needed.^39,42^ In recent studies, PCPs expressed desire for decision support (e.g., patient hand-outs, electronic medical record (EMR) notifications with guideline recommendation updates).^30,43^

#### Other Health System Factors

In three studies (one of good quality), PCPs reported limited time to provide justification for a recommendation to stop screening.^19,38,39^ Clinical reminders^28,35^ and EMR alerts^27^ influenced recommendations in three studies (two of good quality). In two poorer quality studies, PCPs did not consider stool tests and breast screening for older adults worthwhile due to limited cost-effectiveness and unnecessary burden on the health system^14,31^ and costs for the patient was also reason for not recommending screening in two other poorer quality studies.^30,42^ PCPs quality metrics including scores on how well they are screening patients also led them to encouraging screening up to 70 or 75 years, regardless of other factors.^30,31^

### Interaction Between Factors

Table 4 highlights findings from studies that reported the interaction of factors influencing PCPs’ screening recommendations.

#### Age

One vignette study found that PCPs were less likely to recommend colorectal screening to an 80-year-old with congestive heart failure compared to a healthy patient, but this difference was not evident for patients 65 years old.^21^ Another study found that the consideration of health status did not change with increasing age.^34^ The influence of screening history also depended on age, as 53% of PCPs recommended screening for a 75-year-old who had undergone colonoscopy < 5 years ago, but 23% recommended screening for an 80-year-old with the same screening history.^34^ Patient request was also more likely to influence prostate and colorectal screening recommendations for 70-year-olds compared to 90-year-olds.^35^

#### Health

PCPs used average life expectancies of people at that age to guide their colorectal screening decisions for patients in good health, but for patients in poor health, severity of comorbidities and functional status was influential.^24^ PCPs were also more likely to elicit preferences for a patient in fair health compared to a patient in poor health.^24^ Reported difficulty estimating life expectancy did not impact whether life expectancy influenced prostate screening decision-making.^38^

#### PCP Characteristics

Female PCPs were more likely to recommend prostate screening due to patient anxiety compared to male PCPs^41^ and non-black providers were more likely than black providers to report that their patients expected them to continue ordering PSA tests, uncomfortable with the uncertainty of discontinuing screening, and that it takes more time to explain rationale for stopping screening compared to just continuing.^38^ Family and internal medicine PCPs were more likely to be influenced by USPSTF recommendations than obstetrician-gynecologists who were more likely to be influenced by the American Cancer Society or American College of Obstetricians and Gynecologists guidelines.^29^

## DISCUSSION

Our review builds on previous narrative reviews by systematically synthesizing evidence on how patient, clinician, and health system factors influence PCPs’ cancer screening decision-making for older adults. Our findings highlight a complex interplay between patient health factors and clinician psycho-social factors in decision-making for PCPs, and the role of older adults’ screening preferences. US-based research dominates the evidence base, highlighting the need for further research to understand factors influencing PCPs’ decision-making for cancer screening in older adults in non-US countries, including those with national screening programs.

Prior research has posited frameworks for individualizing cancer screening decisions for older adults that consider patient, clinician, and health system factors,^2,47^ and has summarized the evidence and guidelines for cancer screening in older adults.^5,48^ Breslau and colleagues developed the Individualized Decisions for Screening (IDS) framework in 2016, providing a guide for the conceptualization, measurement, and implementation of multi-level interventions to improve the quality of older adult’s screening decisions through consideration of patient, clinician, and health system influences.^2^ Our review provides an up-to-date summary of how PCP decision-making may or may not align with this framework for individualized decision-making.

Regarding patient factors, our review highlights older adults’ preferences particularly influence PCPs’ decision-making, emphasizing the importance of continued efforts to develop and implement tools to support informed choice for screening. Although the gold standard may be that PCPs provide information about the benefits and harms of screening, additional approaches may be helpful such as directly targeting information to older adults, perhaps even before the notion of no longer screening becomes relevant, as previously suggested by Schonberg and colleagues for breast screening.^49^ Older adults have long heard persuasive screening messaging and some have strong positive attitudes towards screening that may outweigh the influence of factors such as limited life expectancy or a clinician’s recommendation.^50^ They may need time to consider tailored information, clarify values, and have ongoing conversations with their doctor and family if desired. Researchers have examined how patient decision aids can improve the quality of cancer screening decisions for older adults and reduce overscreening. For example, Schonberg and colleagues developed a decision aid for women aged ≥ 75 years in the USA, which led to more informed screening decisions and a 9% reduction in the number of women choosing to be screened.^51^ In countries with national screening programs, it may also be useful for information to be provided from screening programs to communicate to individuals before they reach the upper age when screening is no longer recommended. However, further research on the impacts of providing such information outside of US contexts is needed.

Our review also highlighted clinician-level barriers to achieving individualized screening decisions for older adults. Contrary to Breslau and colleagues’ suggested framework,^2^ some PCPs may not make conscious, deliberate screening decisions, may only consider health status in a general sense and life expectancy to varied degrees when making recommendations, and have fears around recommending stopping screening. Other contributors to this challenge are the influence of PCP attitudes and anecdotal experiences on their decision-making, the importance of age-based guidelines, and differences in how they perceive harms such as overscreening. These findings were particularly highlighted in qualitative studies. Continued efforts to develop and implement patient-level interventions such as patient decision aids must also be accompanied by further efforts to support PCPs to provide evidence-based, tailored recommendations to stop screening when it is relevant to do so.

Recommendations to stop screening that incorporate health status (“other health issues should take priority”) may be more acceptable to older adults than recommendations that incorporate life expectancy (“you may not life long enough to benefit”).^52,53^ However, in our experimental scenario-based study, recommendations incorporating life expectancy resulted in reduced intention to screen compared to recommendations incorporating health status.^6^ Specific scripts and strategies co-designed with patients and PCPs to discuss stopping screening in various clinical scenarios have also been developed in the USA, including a briefer statement about stopping, a longer script highlighting reasons for stopping, and a shared decision-making script to support discussion about the benefits and harms,^54^ which could be adapted for use in countries with national screening programs. Further research is needed to test communication strategies that are acceptable to PCPs and effectively support older adults to have a realistic understanding of the benefits and harms of cancer screening, especially for those with strong positive attitudes towards screening.

System-level factors such as time, age-based guidelines, and clinical reminders and electronic medical record alerts are also important influences on PCP recommendations and may form barriers to individualized decision-making. In the USA, it is essential that existing systems are leveraged to better align with the goal of individualized screening decisions. Suggestions include training for PCPs in the use of decision aids, professional organizations expressing support for the use of decision aids, incorporating use of decision aids and life expectancy estimation into electronic medical record alerts,^46,51^ and pre-clinical visits for PCPs to understand patient preferences and understanding ahead of a discussion. In non-US contexts, further research is needed to understand whether and how individualized decisions for cancer screening in older people can be achieved in the context of national screening programs. The design of organized screening programs is likely an important influence on decision-making about cancer screening, for both younger and older adults.

### Limitations

The studies included in our review are limited due to the focus of the literature on the US context, therefore lacking generalizability to other international contexts. Some included studies were also vignette-based, meaning it is difficult to understand whether the factors would have a similar impact in clinical settings. Our review itself was limited as it was not possible to conduct a meta-analysis using any of the quantitative data due to the variability in study designs, measurement, and operationalization of decision-making and influencing factors in the included studies. Extracted data was synthesized narratively, as understanding the relative magnitude of effect sizes for influencing factors was not the aim of this study. This narrative approach allowed for the variation and heterogeneity across studies to be captured, building on the existing narrative reviews on this topic. Strengths of this review were having two independent reviewers conduct screening, data extraction and quality appraisal, and searching studies from grey literature (e.g., theses).

### Conclusion

There are a wide range of patient, clinician, and health system factors that influence PCPs decisions and recommendations for cancer screening in older adults. Decision support should continue to be developed and implemented to support informed choice for older adults and assist PCPs to consistently provide evidence-based recommendations, alongside system changes in electronic medical records, professional organizations, and clinician training. Our findings also highlight the importance of supporting informed choice for younger adults who are beginning to screen, so this information does not become difficult for clinicians to communicate to patients when the benefit/risk trade-off becomes less favorable. Further research is also needed in non-US contexts, especially where national screening programs are implemented, to understand the role of PCPs in older adults’ cancer screening decisions.

### Supplementary Information

Below is the link to the electronic supplementary material.

Supplementary file1 (DOCX 113 KB)

## Data Availability

Data sharing is not applicable to this article as no new data were created or analyzed in this study.
