# Supplementary material for: Factors Influencing Primary Care Practitioners’ Cancer Screening Recommendations for Older Adults: a Systematic Review
Source: J Gen Intern Med. 2023 May 4;38(13):2998–3020. doi: 10.1007/s11606-023-08213-4 (PMC10593684; doi:10.1007/s11606-023-08213-4)
Supplement: Supplementary file 1 — Supplementary file1 (DOCX 113 KB) [file 11606_2023_8213_MOESM1_ESM.docx]

**SUPPLEMENTARY MATERIAL**

**Supplementary Table I.** Search strategy

**Supplementary Table II.** Study quality appraisal using Joanna Briggs Institute critical appraisal checklists

**Supplementary Table III.** Data extraction template

**Supplementary Table III.** Quotes from qualitative studies

**Supplementary Table I.** Search strategy

| OVID Medline & Pre-Medline | Embase | PsycINFO | CINAHL |
| --- | --- | --- | --- |
| 1. geriatrics/  2. geriatric*.tw.  3. older*.tw.  4. elderly.tw.  5. over 65.tw.  6. senior*.tw.  7. pensioner*.tw.  8. veteran*.tw.  9. general practitioners/  10. physicians, primary care/  11. physicians, family/  12. physician*.tw.  13. practition*.tw.  14. provider*.tw.  15. doctor*.tw.  16. clinician*.tw.  17. cancer/  18. cancer.tw.  19. neoplasms/  20. tumo?r*.tw.  21. breast neoplasms/  22. colonic neoplasms/  23. prostatic neoplasms/  24. uterine cervical neoplasms/  25. mass screening/  26. early detection of cancer/  27. (screening* adj3 cancer).tw.  28. mammogra*.tw.  29. PSA test*.tw.  30. prostate-specific antigen.tw.  31. colonoscop*.tw.  32. FOBT.tw.  33. f?ecal occult blood test.tw.  34. pap smear*.tw.  35. pap test*.tw.  36. human papillomavirus.tw.  37. HPV test*.tw.  38. cervical screening test*.tw.  39. decision making/  40. decision*.tw.  41. intention/  42. intention*.tw.  43. attitude/  44. attitude*.tw.  45. view*.tw.  46. perspective*.tw.  47. opinion*.tw.  48. experience*.tw.  49. recommend*.tw.  50. practi?e*.tw.  51. approach*.tw.  52. 1 or 2 or 3 or 4 or 5 or 6 or 7 or 8  53. 9 or 10 or 11 or 12 or 13 or 14 or 15 or 16  54. 17 or 18 or 19 or 20 or 21 or 22 or 23 or 24  55. 25 or 26 or 27 or 28 or 29 or 30 or 31 or 32 or 33 or 34 or 35 or 36 or 37 or 38  56. 39 or 40 or 41 or 42 or 43 or 44 or 45 or 46 or 47 or 48 or 49 or 50 or 51  57. 52 and 53 and 54 and 55 and 56  58. limit 57 to yr=”2000-Current” | 1. geriatrics/  2. geriatric*.tw.  3. older*.tw.  4. elderly.tw.  5. over 65.tw.  6. senior*.tw.  7. pensioner*.tw.  8. veteran*.tw.  9. general practitioners/  10. physicians, primary care/  11. physicians, family/  12. physician*.tw.  13. practition*.tw.  14. provider*.tw.  15. doctor*.tw.  16. clinician*.tw.  17. cancer/  18. cancer.tw.  19. neoplasm/  20. tumo?r*.tw.  21. breast cancer/  22. colorectal cancer/  23. prostate cancer/  24. cervical cancer/  25. cancer screening/  26. mass screening/  27. early cancer diagnosis/  28. (screening* adj3 cancer).tw.  29. mammogra*.tw.  30. PSA test*.tw.  31. prostate-specific antigen.tw.  32. colonoscop*.tw.  33. FOBT.tw.  34. f?ecal occult blood test*.tw.  35. cervical screening test*.tw.  36. pap smear*.tw.  37. pap test*.tw.  38. human papillomavirus.tw.  39. HPV test*.tw.  40. decision making/  41. decision*.tw.  42. intention/  43. intention*.tw.  44. attitude/  45. attitude*.tw.  46. view*.tw.  47. perspective*.tw.  48. opinion*.tw.  49. experience*.tw.  50. recommend*.tw.  51. practi?e*.tw.  52. approach*.tw.  53. 1 or 2 or 3 or 4 or 5 or 6 or 7 or 8  54. 9 or 10 or 11 or 12 or 13 or 14 or 15 or 16  55. 17 or 18 or 19 or 20 or 21 or 22 or 23 or 24  56. 26 or 27 or 28 or 29 or 30 or 31 or 32 or 33 or 34 or 35 or 36 or 37 or 38 or 39  57. 40 or 41 or 42 or 43 or 44 or 45 or 46 or 47 or 48 or 49 or 50 or 51 or 52  58. 53 and 54 and 55 and 56 and 57  59. limit 58 to yr=”2000-Current” | 1. geriatrics/  2. geriatric*.tw.  3. older*.tw.  4. elderly.tw.  5. over 65.tw.  6. senior*.tw.  7. pensioner*.tw.  8. veteran*.tw.  9. general practitioners/  10. physician*.tw.  11. practition*.tw.  12. provider*.tw.  13. doctor*.tw.  14. clinician*.tw.  15. cancer.tw.  16. neoplasms/  17. tumo?r*.tw.  18. cancer screening/  19. (screening* adj3 cancer).tw.  20. mammogra*.tw.  21. PSA test*.tw.  22. prostate-specific antigen.tw.  23. colonoscop*.tw.  24. FOBT.tw.  25. f?ecal occult blood test*.tw.  26. cervical screening test*.tw.  27. pap smear*.tw.  28. pap test*.tw.  29. human papillomavirus.tw.  30. HPV test*.tw.  31. decision making/  32. decision*.tw.  33. intention/  34. intention*.tw.  35. attitude*.tw.  36. view*.tw.  37. perspective*.tw.  38. opinion*.tw.  39. experience*.tw.  40. recommend*.tw.  41. practi?e*.tw.  42. approach*.tw.  43. 1 or 2 or 3 or 4 or 5 or 6 or 7 or 8  44. 9 or 10 or 11 or 12 or 13 or 14  45. 15 or 16 or 17 or 18 or 19  46. 20 or 21 or 22 or 23 or 24 or 25 or 26 or 27 or 28 or 29 or 30  47. 31 or 32 or 33 or 34 or 35 or 36 or 37 or 38 or 39 or 40 or 41 or 42  48. 43 and 44 and 45 and 46 and 47  49. limit 48 to yr=”2000-Current” | S1: (MH “Gerontologic Care”) OR “geriatric*” OR “older*” OR “elderly” OR “over 65” OR “senior*” OR “pensioner*” OR “veteran*”  S2: (MH “Physicians”) OR (MH “Physician Attitudes”) OR “physician*” OR “practition*” OR “provider*” OR “doctor*” OR “clinician*”  S3: (MH “Neoplasms”)OR “cancer*” OR “tumo?r*”OR (MH “Breast Neoplasms”) OR (MH “Colorectal Neoplasms”) OR (MH “Prostatic Neoplasms”) OR (MH “Cervix Neoplasms”)  S4: (MH “Cancer Screening”) OR (screening* N3 cancer) OR “mammogra*” OR “PSA test*” OR “prostate-specific antigen” OR “colonoscop*” OR “FOBT” OR “f?ecal occult blood test*” OR “cervical screening test*” OR “pap smear*” OR “pap test*” OR “human papillomavirus” OR “HPV test*”  S5: (MH “Decision Making”) OR “decision*” OR (MH “Intention”) OR “intention*” OR (MH “Attitude”) OR “attitude*” OR “view*” OR “perspective*” OR “opinion*” OR “experience*” OR “recommend*” OR “practi?e*” OR “approach*”  S6: S1 AND S2 AND S3 AND S4 AND S5 |

| **Checklist for Cross-sectional Studies (9 items)** | | | | | | | | | | |
| --- | --- | --- | --- | --- | --- | --- | --- | --- | --- | --- |
| **Study  (author, year)** | **CHECKLIST ITEMS** | | | | | | | | | **OVERALL APPRAISAL** |
|  | **Was the sample frame appropriate to address the target population?** | **Were study participants sampled in an appropriate way?** | **Was the sample size adequate?** | **Were the study subjects and setting described in detail?** | **Was the data analysis conducted with sufficient coverage of the identified sample?** | **Were valid methods used for the identification of the condition?** | **Was the condition measured in a standard, reliable way for all participants?** | **Was there appropriate statistical analysis?** | **Was the response rate adequate, and if not, was the low response rate managed appropriately?** |  |
| Boone 2018 | ? | + | + | + | + | - | + | ? | + | Low |
| Haas 2017 | ? | ? | - | + | ? | + | + | + | ? | Moderate |
| Haggstrom 2013 | + | + | + | + | ? | ? | + | ? | + | Low |
| Heflin 2006 | + | + | + | + | + | + | + | + | + | Low |
| Kadaoui 2012 | + | + | - | + | ? | + | + | + | + | Low |
| Kahi 2009 | ? | + | - | + | ? | - | + | + | + | Moderate |
| Kistler 2018 | - | ? | - | + | ? | ? | + | + | + | Moderate |
| Konety 2006 | + | ? | ? | + | ? | - | + | + | ? | Moderate |
| Konety 2009 | + | ? | ? | ? | ? | + | + | + | + | Moderate |
| Leach 2012 | + | + | + | + | ? | + | + | + | ? | Low |
| Lewis 2008 | - | _ | - | ? | ? | + | + | ? | + | High |
| Lewis 2013 | + | + | + | + | ? | ? | + | ? | + | Low |
| Pollack 2012 | - | ? | - | + | ? | - | + | + | + | Moderate |
| Pollack 2017 | + | + | ? | + | ? | - | + | + | ? | Low-moderate |
| Radhakrishnan 2018 | + | + | ? | + | ? | ? | + | + | ? | Low |
| Ruff 2005 | ? | ? | - | - | ? | - | + | - | + | High |
| Sharp 2005 | ? | ? | - | - | ? | - | + | - | + | High |
| Siembida 2017 | + | + | ? | + | ? | - | + | + | + | Low |
| Sifri 2019 | - | ? | - | - | ? | - | + | - | ? | High |
| Yasmeen 2012 | + | + | + | + | ? | + | + | + | - | Low |
| Walters 2011* | ? | ? | - | + | ? | - | + | ? | - | High |

**Supplementary Table II.** Study quality appraisal using Joanna Briggs Institute critical appraisal checklists

*Mixed methods study

(+) met criteria, (-) did not meet criteria, (?) unclear

Low: ≤2 domains of concern or ≤3 domains unclear, moderate: 3-4 domains of concern where at least one domain is not fulfilled (-) or high: ≥5 domains of concern

| **Checklist for Qualitative Studies (10 items)** | | | | | | | | | | | |
| --- | --- | --- | --- | --- | --- | --- | --- | --- | --- | --- | --- |
| **Study  (author, year)** | **CHECKLIST ITEMS** | | | | | | | | | | **OVERALL APPRAISAL** |
|  | **Is there congruity between the stated philosophical perspective and the research methodology?** | **Is there congruity between the research methodology and the research question or objectives?** | **Is there congruity between the research methodology and the methods used to collect data?** | **Is there congruity between the research methodology and the representation and analysis of data?** | **Is there congruity between the research methodology and the interpretation of results?** | **Is there a statement locating the researcher culturally or theoretically?** | **Is the influence of the researcher on the research, and vice- versa, addressed?** | **Are participants, and their voices, adequately represented?** | **Is the research ethical according to current criteria or, for recent studies, and is there evidence of ethical approval by an appropriate body?** | **Do the conclusions drawn in the research report flow from the analysis, or interpretation, of the data?** |  |
| Austin 2021* | + | + | + | + | + | ? | - | + | + | + | Low |
| Enns 2021 | ? | + | + | + | + | + | ? | + | + | + | Low |
| Lewis 2009 | + | + | + | + | + | - | - | + | + | + | Low |
| Oshima 2021 | ? | + | + | + | + | - | - | + | + | + | Low-moderate |
| Park 2021 | ? | + | + | + | + | - | - | + | + | + | Low-moderate |
| Rowe 2021 | ? | + | + | + | + | ? | - | + | + | + | Low |
| Schoenborn 2020a | + | + | + | + | + | + | - | + | + | + | Low |
| Schoenborn 2020b | ? | + | + | + | + | - | - | + | + | + | Low-moderate |
| Schonberg 2006 | ? | + | + | ? | + | - | - | + | + | + | Moderate |
| Walters 2011+ | ? | + | + | + | + | - | - | + | + | + | Low-moderate |

*Mixed methods study

(+) met criteria, (-) did not meet criteria, (?) unclear

Low: ≤2 domains of concern or ≤3 domains unclear, moderate: 3-4 domains of concern where at least one domain is not fulfilled (-) or high: ≥5 domains of concern

**Supplementary Table III.** Data extraction template

| **Study characteristics and eligibility** | | | | | | | | | | |
| --- | --- | --- | --- | --- | --- | --- | --- | --- | --- | --- |
| **Study & year** | **Study design** | **Country** | **Participant description** | **Study aim** | **Type of screening** | **Sample size** | **Response rate** | **Eligible?** (1st author) | **Eligible?** (2nd author) | **FINAL DECISION** |
| Sifri 2019 | Cross-sectional, vignettes | United States | community providers (physicians, fellows, residents, physician assistants, nurse practitioners) – Philadelphia | To assess current PCP approaches to breast and colorectal cancer (CRC) screening for patients ages 75+ years | Colorectal Breast | 51 | 46.40% | Yes | Yes | YES |

| **Quantitative studies** | | | | | | | | | |
| --- | --- | --- | --- | --- | --- | --- | --- | --- | --- |
| **Study & year** | **Study design** | **Key outcomes** | **Analysis** | **Factors** | | | | | |
|  |  |  |  | ***Patient demographic*** | ***Patient health*** | ***Patient psycho-social*** | ***Clinician characteristics*** | ***Clinician psycho-social*** | ***System*** |
| Sifri 2019 | Cross-sectional, vignettes | Current approaches and practices concerning cancer screening in older adults | Descriptive statistics |  | Ranked the 3 most important factors that determine whether to order cancer screening for someone age >75. The top factors were life expectancy (1st - 84.3%) and severity of medical conditions (3rd) (70.6%). | Patient preference 2nd most important factor determining whether to order screening for someone age >75 (82.4%) |  |  | 78.4% of PCPs did not use tools to assist with decision making.  They would find methods for estimating life expectancy helpful (60.8%), decision aids for providers to estimate benefits/harms of cancer screening (64.7%), and decision aids for patients that present personalized benefits/harms of cancer screening (66.7%) |

| **Qualitative studies** | | | | | | | | | |
| --- | --- | --- | --- | --- | --- | --- | --- | --- | --- |
| **Study & year** | **Study design** | **Key outcomes** | **Analysis** | **Factors** | | | | | |
|  |  |  |  | ***Patient demographic*** | ***Patient health*** | ***Patient psycho-social*** | ***Clinician characteristics*** | ***Clinician psycho-social*** | ***System*** |
| Schonberg 2006 | Semi-structured interviews | Counselling about breast screening to women aged ≥80 | Iterative process following standard techniques | Patient age.  Three commented that they were less likely to recommend screening to patients as they aged into their late 80s or 90s. | Patient’s health, functional status, and life expectancy. ‘‘If I think that someone has 1 to 3 years to live it is preposterous to start screening. If someone at the other end is incredibly healthy and may have 15 to 20 years ahead of them, then at least theoretically it makes more sense.’’ Eight said the availability of acceptable treatments for elderly women influenced their recommendations | If patients had a preference about screening they followed the patient’s preference.  Six described that a longstanding doctor-patient relationship and trust can facilitate discussions. ‘‘It has a lot to do with trust, especially if someone is accustomed to having regular mammograms done and you are recommending not [to] do it anymore.’’ |  | Difficulties when discussing stopping screening. Six commented that discussing stopping can be uncomfortable, ‘‘it is very hard for me to say you do not need a mammogram, you are not going to benefit. . .so sometimes even though I know that it does not make sense, I will still do it.’’ | Six requested more data about the risks/benefits for women aged ≥80: ‘‘it would be nice if we had better studies, better guidelines… that might make it a little easier.’’  Six described difficulty for patients to understand the risks and 3 said this discussion can take a great deal of time during an already busy clinic. ‘‘there is a magical thinking that goes on about cancer screening with people. They feel that if they do it, it protects them. I do not disavow people of that just because it is too time consuming.’ |

**Supplementary Table III.** Quotes from qualitative studies

| **Factor** | **Quotes** |
| --- | --- |
| **Patient demographic and health characteristics** | |
| Age | **For**: “I’ll just do 75 for everything”^29^  **Against**: “I try not to use age as the end-all be-all cut off.”^20^  **Differences**:  “I have a tighter [threshold] for colonoscopy…. Generally, I don’t think of it for anyone … after the age of 70 but if I am thinking about some kind of colon screening, then I’m thinking something less invasive so then I go for Cologuard.”^29^  “I usually stop screening for colon cancer at 80…I usually do mammograms until about 85”^29^  Breast: “Mammography is a little different…I will do [mammograms] at any age because the risks are so minimal”. PCa - “because it’s just bloodwork”. CRC - “I think the recommended age to stop doing just routine screening colonoscopies is about 75 so I stick with that”^24^ |
| Life expectancy | **For**:  "The whole thing with colon cancer screening is that you’re trying to find something before and make a difference 5-10 years down the road. If you don’t have 5, 10 years then what’s the point?"  “I agree with an approach more towards [using] life expectancy than just age-based screenings because obviously there [are] very sick 55-year-olds [for whom screening] could not make sense.”^34^  “I find that the 10-year life expectancy is a really helpful guidepost for me…as an evidence-based recommendation to help me to be able to say with confidence: ‘no, you shouldn’t get this screening’.”^34^  ‘‘If I think that someone has 1 to 3 years to live it is preposterous to start screening. If someone at the other end is incredibly healthy and may have 15 to 20 years ahead of them, then at least theoretically it makes more sense.’’^35^  “Certainly if they are unfit and have limited life expectancy, then it's inappropriate, but if they are totally fit and got another 15–20 years you can't justifiably turn them down.”^39^  “If the patient is too sick to have a colonoscopy no matter what… then don’t offer a Cologuard. Because what are you gonna do if the guy is 90-years-old and you offer Cologuard and it’s positive, what do you do then?” “I used to have a lady with emphysema was on oxygen who’s in her late 70 s and… she wanted colon cancer screening and we did Cologuard.”^29^ **Against**:  “That’s really a horrible message to give to people that, “Oh you’re going to die soon so you really don’t need that.” And so if women want to have the imaging then I think that they should and we don’t have the information on older people except to say when people get breast cancer when they’re older it usually grows a little bit slow.”^13^  “I’m not convinced that any of those predictive algorithms are accurate enough to apply to a single person in clinical care… you can look at a population of people and say X percent will live Y years from today but it’s incredibly risky and foolhardy to bring that to a single person.”^34^  “The thing about using life expectancy is it feels calculated and … sort of challenges my humanity and my human connection with someone.”^34^  “If 10 years is the rule then I don’t think any of my patients are gonna live for 10 years… and [that] instinctively makes me a little bit uncomfortable….it feels like quitting but at the same time I realize that it’s realistic.”^34^  “To be quite honest as a black physician and enduring or experiencing my own level of racism… I automatically perceive [a prognostic tool] as a negative, as maybe another tactic to potentially not give minorities the care that they need because there’s gonna be bias. These biases exist now amongst minorities… somebody may say: ‘well, you know this black person - they’ll be dead in 5 years so we don’t need to screen them anyway… because they are gonna die anyway because that’s what the guidelines say’”^34^  “My fear is that people in the decision-making positions are going to influence how those [life expectancy prediction] programs get written…Some people are gonna build the bias in so that everybody will get screened and tested. Other people are gonna build the bias in so that the least amount of money is gonna get spent so we can spread healthcare among more people.”^34^  **Difficulties**:  “You get into the gray area because nobody knows. We don’t have a crystal ball.”^19^ |
| Functional status | “Her son has to take off of work …every time she goes anywhere… For the colonoscopy, people have to take off the whole day.”^29^ |
| Co-morbidities | “I am a big fan of the FIT test… the risk benefit ratio is much better with the FIT test particularly in older patients. You have patients taking diuretics or patients that are a little hypotensive anyway, to think about putting them through a colonoscopy prep gives me pause. So, I would say if I have somebody that I’m on the fence with and I can do a FIT test instead of a colonoscopy I would encourage that for them.”^29^ |
| Risk | *“*Age is the biggest risk factor”^39^ |
| Cognitive impairment | “Consenting to the screening process includes being able to understand the consequences of a diagnosis of cancer. I think if they can't consent we shouldn't make them come.”^39^ |
| **Patient psycho-social** | |
| Patient preference | **For screening:**  “Now the annual mammogram [letter] that I learn tell people that they are overdue for their mammogram and my patients either will go around me to schedule which they can do or they will alternatively bring me the letters from radiology and say, “This says I’m overdue,” and that’s the whole of the conversation that fairly prompts me to say the same things that I’ve said.”^13^  “…if she came in saying I want to do everything I possibly can to stay alive because I’m responsible, but if she was already seeming like, quality of life is not that great and look at her cardiovascular risk factors.”^19^  “I mean it’s totally their decision. My role is just to remind them and to advise them.”^19^  “Our obligation is to give them the data. Give it to them in a way they can understand. If they don’t have any problems understanding why we’re talking about this and then let them make the decision themselves. It’s not our decision to make. It’s theirs.”^19^  “I get patients that do ask about [stool tests], and I tell them that it’s your personal preference.”^29^  “So, the Cologuard we use quite a bit mostly in patients who are reluctant to have an invasive procedure.”^29^  “Patient is seriously interested in it. Recognizes the downsides and false alarms…Patient preference matter a lot to me.” [PSA] “some patients are very insistent or expectant that they want to continue to get a PSA.”^14^  “It is not necessarily evidence-based medicine, but if people feel like taking good care of their health and having their doctor care about that means they get an annual mammogram, I’m fine with that…. For somebody that I didn’t think [mammography] was indicated I still wouldn’t say no.”^33^  “I’ll always default to patient preference so long as there’s been a conversation where I feel it’s informed consent.”^34^  *“*Quite a lot of elderly patients ask if they can come back for screening when it ceases.”^39^  **For treatment:**  “I try talking with [patients] about whether, if we find something, would you want to know? Would you want any interventions, knowing [that] chemo and radiation and survey are very invasive? …If we find something, would you want to know and have interventions [done], versus do you just want to know, versus do you not want to know, in which case we don’t need to get the screening?”^20^ |
| Patient-provider relationship | “For any age, the better I know a patient the better it is because I know if they have declined other screening tests in the past… but if they are the type of person that always wants to come in and get their things done, then I often will bring up [screening].”^20^  ‘‘It has a lot to do with trust, especially if someone is accustomed to having regular mammograms done and you are recommending not [to] do it anymore.’’^35^ |
| Family support | *“*I might look at also their family support. Like if they have a husband who’s going to help with the prep or if she lives alone.”^19^  “But, family and even their kids [are important]. I get older folks where their sons and daughters are the ones telling them they need a colonoscopy more than they’re wanting to have one. I’m not saying that’s something that makes sense, but it’s something that gets considered.”^19^ |
| Anecdotes | “I feel like I am less likely to impact a woman’s decision to have a mammogram than her sister who caught a breast cancer on it or her neighbor who is convinced that her mammogram gave her cancer. That stuff is hard to break through.”^33^ |
| **Clinician psycho-social** | |
| Weighing benefits/ harms | **Breast**:  “Is it worthwhile if three other women got screened for breast cancer and might have issues that come up as a result but you find an early breast cancer and then don’t have the mortality morbidity associated with that down the road?…Does this person’s harm outweigh this [other] person’s benefit, especially if you don’t know which one is actually going to be the beneficiary?”^34^  “Breast is the easiest to treat because it’s a very superficial organ and you don’t have to muck with anything else essential especially when the tumor is small”^24^  “the breast cancers that typically are presenting that late in life tend not to be very aggressive or lethal in my mind so having to go through things at 80 … even something as simple as a routine biopsy, might cause more complications and strife than in the younger population.” ^20^  **Prostate**:  “The PSA by itself has a low risk but … if you have an abnormal PSA then all the urologists around here are gonna recommend a biopsy and that’s not such a benign procedure anymore”^24^  **Colorectal**:  “In terms of colon cancer screening, [colon cancer] can be a rapidly moving cancer from what I understand so … I feel like he should still be checked for it because we can still do something”^24^  "I mean, if someone is 80 and you recommend a colonoscopy and then their bowel got [perforated]. What was I thinking, 80 years old, doing this colonoscopy?"^19^  “I think we always have to screen for things that are malicious and are reversible like colon cancer. I donʼt think you ever overscreen for CRC because itʼs something very preventable and malignancy can be very bad.” ^34^  **Difference between types**:  “Mammography for screening is not a great technology versus the standard with colonoscopy, and colon cancer screening is much better, so I tend to push with that a little bit a little more”^24^  “When you think about mammograms for cancer screening, that’s less invasive than … a colonoscopy. You don’t have the same type of surgical risk or other risks of pain or discomfort. With a mammogram, it’s uncomfortable but they’re just pulling on the skin and getting images it’s not a lot of radiation either, so it’s less invasive. I’m less picky about that”^24^  **Overscreening:**  “We order so many labs and testing. I think we deﬁnitely overscreen patients unnecessarily.” ^34^  “We are looking for smaller and smaller lesions, we are picking up little bits of micro calcification, we are bringing women back for repeat biopsies... for the detection of smaller and smaller lesions that may never even have been invasive cancer. And even if they had been invasive cancer they might never have threatened their lives.” ^39^  **Treatment**:  "But she’s not a candidate for any kind of surgery and what’s the benefit of removing a polyp, you know?"^19^  **Not conscious decision:**  "You know my gut feeling about this person is, you know, they wouldn’t benefit from it."^19^  “I referred her for a colonoscopy…. Now I am thinking that the decision was incorrect…. I am not sure how I came to that decision right now.” ^33^  “Sometimes decisions are made in a hurry in a rushed clinic, and they are not really thought through. It would be foolish for me to pretend that every decision I make on these patients is the product of a rational thought process and shared decision making.” ^33^  **Other:**  “there are a lot more variations from one person to another. A lot more to consider. It’s not just a matter of how old they are and what’s their family history”^19^  ”I kind of believe two physicians who basically screen annually until death…So if we have a pretty decent detection test and breast biopsy is a relatively benign procedure, not super morbid, why not just do it?”^13^ |
| Fear | “I mean god forbid you tried to convince them to be screened bi-yearly and then they have something on their mammogram and they didn’t get it yearly, like the hospital recommended. You know, like if that ever happened the doctor would be in a terrible position, even though that’s what the guidelines say.”^13^ |
| Familiarity | “We are not using the [newer stool tests] here just quite yet. I guess they’re not that common so I’m not that familiar with them. I’m still gonna [use] classic colonoscopy. I do know that there’s new technology… but we are not that familiar so I don’t know how reliable it is. I guess I need to do more research on that.”^29^ |
| Test effectiveness | “[Cologuard] picks up like 90% of colon cancer but it only picks up about 15% of advanced polyps which is really what you want to find because you don’t really care once they’ve had cancer already well then you’re sort of behind the 8 ball. You’re really trying to pick these up the advanced adenoma stage where they can remove the adenoma and hopefully prevent them from getting cancer.”^29^ |
| Resistance to change | “For a 77 otherwise in good health, generally I would do test….But generally I won’t have a long discussion in terms of whether they should do it or not.” ^14^ |
| Difficulty of discussion | ‘‘it is very hard for me to say you do not need a mammogram, you are not going to benefit… so sometimes even though I know that it does not make sense, I will still do it.’’ ^35^ |
| **Health system** | |
| Guidelines | “I think that this is a really difficult area because even though I feel pretty confident in the guidelines and the data that mammograms should be every two years, our patients get a letter reminding them that they should have their yearly mammogram. I think that that creates a lot of confusion.”^13^  **Scepticism towards guidelines**:  “I don’t want to see us moving to where people who control the money simply say—ain’t paying for that because based on this actuarial table you are likely to die in the next 7.1 years.”^34^  “Anytime science makes a dramatic change in their recommendations from PSA screening every man at age 40 to this sort of casual ‘if you want, you can start talking about it at age 55, but you donʼt really have to.’ I am uncomfortable with that…My concern is we might end up back peddling…years later we realize we should have been doing PSA.” ^34^  “I think it's not necessarily the women who most need screening who are the ones who get it, it's the ones who are aware of it, who are more educated, more well read who are then able to access that service.”^39^  “I’ve been here a long time I’ve learned that certain providers are very set in their ways and don’t want intervention”^13^  **Need for better guidelines**:  “it would be nice if we had better studies, better guidelines. . .that might make it a little easier.’’ ^35^ |
| Time / workload | ‘‘there is a magical thinking that goes on about cancer screening with people. They feel that if they do it, it protects them. I do not disavow people of that just because it is too time consuming.’ ^35^  “Our workload in general practice is getting more and more. I'm certainly reticent about putting in another visit to the GP to discuss something like that.” ^39^ |
| EMR alerts | “You may just click off [the alert], like: ‘Oh you are due for a mammogram’, and order it without really considering whether or not it’s appropriate.” ^33^  “What happened in all honesty is I probably would offer him a screening… but it disappeared from the prompt.” ^33^  “Even if I thought you were due for this [screening]… if [an EMR alert] doesn’t pop up, then I say: ‘OK it must have been done or must be this patient doesn’t need it’… I trust the power to be to have the system set to not miss anything or not over prompt.” ^33^ |
| Decision aids | “There’s a couple of pretty decent decisions aids out there for breast cancer screening…Having that integrated in to the EMR in a meaningful way that could be very useful.” ^13^ |
| Cost / resources | “If people are struggling financially and looking for things not to do, I certainly put it out there… as optional.” ^20^  “Current pricing [of] Cologuard is almost as expensive as a colonoscopy…if it comes up abnormal, then you still have to go back to the invasive diagnostic test so you’re spending more money that way.” ^29^  “If we are giving the option of something unproven, and potentially unnecessary, we are diverting resources away from, say, physiotherapy for stroke patients.” ^39^ |
| Quality metrics | “We get scored on how well we are screening patients… up to the age of 70. If they are [under] 70 and I don’t think they should have any more colorectal cancer screening I might still ask them to get a FIT so that my quality numbers are appropriate rather than having them just not be tested at all.” ^29^ |
| Not returning tests | “It’s hard to get people to do FIT tests; we hand out a lot of FIT tests that never get returned.” ^29^ |
| Specialists | “Some patients seem to be on sort of BCa [screening] auto pilot…the BCa screening folks have taken over that and I tend to have less of a role. The same thing can be with colonoscopy, some people have an outside gastroenterologist who has gotten them set up on a 5-year screening plan and I’m just sort of along for the ride.” ^33^ |
